# Association of glucagon-like peptide-1 receptor agonists with cardiac arrhythmias in patients with type 2 diabetes or obesity: a systematic review and meta-analysis of randomized controlled trials

**DOI:** 10.1186/s13098-022-00970-2

**Published:** 2022-12-26

**Authors:** Sijin Wu, Wenzhao Lu, Zhongli Chen, Yan Dai, Keping Chen, Shu Zhang

**Affiliations:** State Key Laboratory of Cardiovascular Disease, National Center for Cardiovascular Diseases, Arrhythmia Center, Fuwai Hospital, Chinese Academy of Medical Sciences & Peking Union Medical College, No.167, Beilishi Road, Xi Cheng District, Beijing, 100037 China

**Keywords:** GLP-1 RAs, Arrhythmias, Atrial fibrillation, Diabetes mellitus, Obesity

## Abstract

**Background:**

Glucagon-like peptide-1 receptor agonists (GLP-1 RAs) have been highly recommended for glycemic control and weight reduction. However, evidence has accumulated that GLP-1 RAs treatment is related to an increase in heart rate, which could potentially induce cardiac arrhythmias. This study aims to investigate the association of GLP-1 RAs therapy with incident arrhythmias in diabetic and obese patients.

**Methods:**

MEDLINE, EMBASE, Cochrane Library, and ClinicalTrials.gov were systematically searched from inception up to May 25, 2022. Randomized controlled trials (RCTs) comparing GLP-1 RAs with placebo or active control for adults with type 2 diabetes or obesity were included. The outcomes of interest were prespecified as incident atrial fibrillation (AF), atrial flutter (AFL), ventricular arrhythmias (VAs), and sudden cardiac death (SCD). Mantel-Haenszel relative risk (MH-RR) with a corresponding 95% confidence interval (95% CI) was estimated using a fixed-effects model.

**Results:**

A total of 56 RCTs involving 79,720 participants (44,028 GLP-1 RAs vs 35,692 control: mean age 57.3 years) were included from 7692 citations. GLP-1 RAs use overall did not significantly increase the risk of AF (RR 0.97, 95% CI 0.83–1.12), AFL (RR 0.83, 95% CI 0.59–1.17), VAs (RR 1.24, 95% CI 0.92–1.67), and SCD (RR 0.89, 95% CI 0.67–1.19), compared with controls. In further subgroup analyses, we observed an increasing trend toward incident AF with dulaglutide (RR 1.40, 95% CI 1.03–1.90) while an inverse trend with oral semaglutide (RR 0.43, 95% CI 0.21–0.87). Additionally, higher doses of GLP-1 RAs (RR 1.63, 95% CI 1.11–2.40) and higher baseline BMI (RR 1.60, 95% CI 1.04–2.48) might significantly increase the risk of VAs. No significant differences were identified in other subgroup analyses.

**Conclusions:**

GLP-1 RAs therapy was not associated with an overall higher risk of arrhythmias, demonstrating an assuring cardiovascular safety profile. Further studies are required to determine whether the potential antiarrhythmic or arrhythmogenic effect of GLP-1 RAs is drug-specific and varies from doses or baseline BMI.

***Trial registration***: PROSPERO Identifier: CRD42022339389.

**Supplementary Information:**

The online version contains supplementary material available at 10.1186/s13098-022-00970-2.

## Background

Glucagon-like peptide-1 receptor agonists (GLP-1 RAs) are relatively novel anti-hyperglycemic drugs that mimic the effects of endogenous GLP-1 and contribute to favorable glycemic control and weight loss. Evidence has accumulated that GLP-1 RAs could be also associated with improved cardiovascular outcomes and a reduction in major adverse cardiovascular events (MACEs) [1–3]. They are now highly recommended for patients with type 2 diabetes mellitus (T2DM) and obesity, particularly for those at high risk for cardiovascular diseases. Considering the widespread use of these drugs, their cardiovascular safety and potential adverse effects warrant attention.

Multiple studies have indicated that treatment with GLP-1 RAs could slightly increase heart rate (HR) [4–6], which has been observed across different populations in both short-acting and long-acting agents, implying a potential class effect of these emerging drugs [4]. This observation also raised some concerns about the potential adverse effect of GLP-1 RAs on the risk of incident cardiac arrhythmias.

Cardiac arrhythmia, commonly known as atrial fibrillation (AF), atrial flutter (AFL), and ventricular arrhythmias (VAs), is a severe and prevalent condition, representing a major health burden worldwide. Arrhythmia events had been reported in numerous randomized controlled trials (RCTs) involving GLP-1 RAs, especially in several large-scale cardiovascular outcome trials (CVOTs) [7–12]. REWIND trial revealed that patients with dulaglutide had a significantly higher incidence of AF than patients with placebo (nearly 1.8% vs 1.2%) [8]. Consistently, an aggregate analysis of patient-level data from Harmony registration studies with albiglutide treatment demonstrated a significant increase in the risk of AF and AFL [13]. In other CVOTs (Harmony Outcomes trial [7], LEADER trial [9], ELIXA trial [10], PIONEER-6 trial [11], and SUSTAIN-6 trial [12]), the incidence of AF between GLP-1 RAs and control was not significantly different. LEADER trial showed that liraglutide had a tendency to increase VAs, but the result was not statistically significant [9]. To date, the effects of GLP-1 RAs treatment on arrhythmia events remain uncertain. Given the serious hazard of arrhythmias, it is important to figure out the association between GLP-1 RAs and arrhythmias.

Therefore, we conducted a systematic review and meta-analysis of RCTs to investigate the association between GLP-1 RAs therapy and the risk of arrhythmias. Furthermore, we also examined whether the risks differed across different drug types, treatment dosage, follow-up duration, study designs, and baseline body mass index [BMI].

## Methods

This systematic review was conducted according to the Preferred Reporting Items for Systematic Reviews and Meta-Analyses (PRISMA) guidelines [14]. The protocol of this study had been prospectively registered on the PROSPERO website (Number CRD42022339389).

### Data sources and searches

A literature search was systematically performed through PubMed, Embase, Cochrane library, and ClinicalTrials.gov. from their inception up to May 25, 2022, without language restrictions. We further extended the search to reference lists of published trials and reviews to maximize the identification of all relevant trials about GLP-1 RAs use. A detailed search strategy is available in Additional file 1: Method S1. The database search was performed on May 26, 2022.

### Study selection and main outcomes

According to the predefined inclusion and exclusion criteria (available in Additional file 1: Table S1), two independent reviewers (SW and WL) screened the titles and abstracts of all records, and then evaluated the full texts of eligible studies. Conflicts over study inclusion were resolved through consensus or discussion with other team members. Only RCTs comparing GLP-1 RAs with placebo or active control in adults with diagnosed T2DM or obesity were included. We excluded trials if they: compared different GLP-1 RAs individuals, compounded preparations of GLP-1 RAs with other drugs(e.g., IDegLira), had a short follow-up duration (< 24 weeks), failed to report arrhythmia endpoints, or reported zero events in both experimental and controlled arms. Exploratory or post-hoc analyses of existing trials were also excluded.

The main outcomes of interest were incident cardiac arrhythmias. We selected several frequent types of arrhythmias, including AF, AFL, and VAs (e.g., ventricular extrasystoles, ventricular tachycardia, ventricular fibrillation, ventricular flutter, and torsades de pointes). Since most SCD cases are closely linked to the aforementioned severe VAs, we also included incident SCD as part of the outcome. The SCD variable was predefined as cardiac arrest, sudden death, and sudden cardiac death*.*

### Data extraction and quality assessments

Two investigators (SW and WL) separately extracted relevant data that were confirmed by the senior author (KC). Retrieved data included study characteristics (study names, registration number, year of publication, population size), characteristics of participants (mean age, male proportion, baseline BMI, and glycated hemoglobin [HbA1c]), interventions and comparations (type and dosage of GLP-1 RAs, comparator drugs), and outcome data (incidence of arrhythmias or SCD events). Almost all included studies had reported arrhythmia events as severe adverse events (SAEs) and did not report relevant data in the trial publication, therefore, we searched the results section (adverse event) of the study page on ClinicalTrials.gov or EudraCT.

Two investigators (WS and WL) independently assessed the quality of these eligible RCTs using the revised Cochrane risk-of-bias tool (RoB2) for RCTs [15]. Disagreements were resolved by consensus.

### Data synthesis and analysis

Mantel-Haenszel relative risk (MH-RR) with a corresponding 95% confidence interval (95% CI) was estimated to present the incidence of arrhythmias. RR > 1 would favor that GLP-1 RAs increase the risk of the events compared with controls. For studies with a zero count, 0.5 was added to all cell frequencies of these studies. The cumulative incidence of each endpoint was expressed as events per 10,000 patient-years. Heterogeneity was evaluated by using Cochrane’s Q tests and *I*^2^ statistics. It was considered statistically significant that *P* value < 0.1 for the Q test. For *I*^2^ statistics, heterogeneity was regarded as high if *I*^2^ value was > 50% [16]. We used a random-effects model when there was high heterogeneity between studies. Otherwise, a fixed-effects model was used. We performed sensitivity analyses with a fixed-effects model by omitting each eligible trial iteratively. Funnel plots and egger’s test for each outcome were examined to assess possible publication bias.

Subgroup analyses were conducted to further investigate the potential risk factors of GLP-1 RAs use on arrhythmias. Subgroup categories were specified as follows: (1) different GLP-1 RAs drugs, including albiglutide, dulaglutide, exenatide, liraglutide, lixisenatide, or semaglutide (since semaglutide is administered subcutaneously or orally, we divided it into two groups); (2) study designs, including CVOT or non-CVOT; (3) follow-up duration (≤ 52 weeks or > 52 weeks); (4) baseline BMI, including higher than the average weight of all included trials, or lower; (5) different dosages (higher or lower). Based on current clinical practice, we considered high dosages of GLP-1 RAs as greater than or equal to: liraglutide, 1.8 mg daily; albiglutide, 50 mg weekly; dulaglutide, 1.5 mg weekly; oral semaglutide, 7 mg daily; subcutaneous semaglutide, 1.0 mg weekly. Other dosages of these drugs were considered low. Exenatide and lixisenatide were excluded from the dosage-effect assessment since they were single dosage. We performed subgroup analyses for each outcome mentioned above in turn.

Data analyses were performed using R (version 4.1.2, 2021-11-01) with the “meta” package (version 5.2-0). All statistical tests were two-sided, and *P* values < 0.05 were considered statistically significant.

## Results

### Characteristics of included studies

**Fig. 1:**
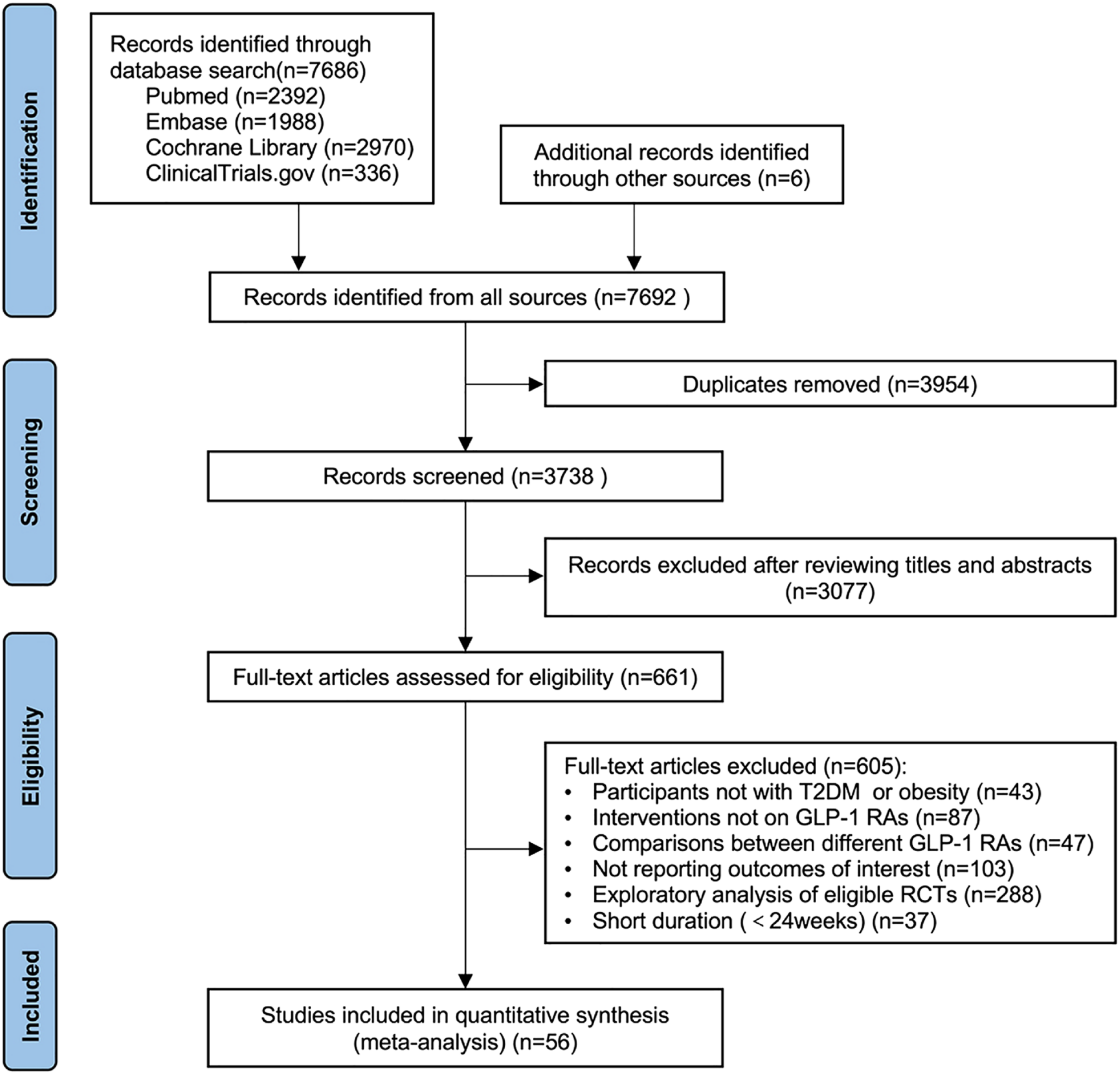
PRISMA flow diagram. Abbreviations: *GLP-1 RAs* glucagon-like peptide 1 receptor agonists, *RCTs* randomized controlled trials, *T2DM* type 2 diabetes mellitus

After in-depth literature selection, we included 56 RCTs (including 1 unpublished trial) in quantitative synthesis from a total of 7,692 citations (Fig. 1). GLP-1 RAs used were albiglutide (9 studies), dulaglutide (10 studies), exenatide (7 studies), liraglutide (10 studies), lixisenatide (5 studies), oral semaglutide (5 studies), and sc semaglutide (10 studies). These studies comprised a total population size of 79,720: 44,028 (55.2%) patients treated with GLP-1 RAs and 35,692 (44.8%) controls. The participants were all adult patients with T2DM or obesity. The mean age, baseline BMI, and HbA1c were 57.3 ± 4.8 years, 32.4 kg/m^2^, and 8.0%, respectively. Across all included studies, the median follow-up duration was 52 weeks (ranging from 24 weeks to 5.4 years), providing 166,579 patient-years. The characteristics of included studies and participants are listed in Table 1 and Additional file 1: Table S2.

**Table 1:** Baseline characteristics of studies and participants included

Trials, year	Drug	Size	Age (years)	BMI (kg/m^2^)	HbA1c,%	Follow-up duration	Outcomes of interest
HARMONY 1, 2014	Albiglutide	301	55.0	34.1	8.1	52 weeks	AF
HARMONY 3, 2014	Albiglutide	1012	54.5	32.7	8.1	104 weeks	SCD
HARMONY 4, 2014	Albiglutide	745	55.5	33.1	8.3	52 weeks	AF/AFL/VAs
HARMONY 8, 2014	Albiglutide	495	63.3	30.4	8.2	52 weeks	AF/AFL/VAs
Rosenstock et al., 2014	Albiglutide	566	55.6	NA	8.5	26 weeks	AF/VAs
HARMONY 5, 2015	Albiglutide	663	55.2	32.2	8.2	52 weeks	AF
HARMONY 2, 2016	Albiglutide	301	52.9	33.9	8.2	52 weeks	AF
Harmony Outcomes, 2018	Albiglutide	9432	64.1	32.3	8.7	78 weeks	AF/AFL/VAs/SCD
Rosenstock et al., 2020	Albiglutide	813	58.1	32.1	7.7	26 weeks	AF/AFL
Ferdinand et al., 2014	Dulaglutide	755	56.5	33.0	7.9	26 weeks	VAs
AWARD-3, 2014	Dulaglutide	807	55.6	33.3	7.6	52 weeks	AF
AWARD-5, 2014	Dulaglutide	1098	54.0	31.3	8.1	52 weeks	AF/SCD
AWARD-2, 2015	Dulaglutide	807	56.6	31.6	8.1	78 weeks	VAs
AWARD-4, 2015	Dulaglutide	884	59.4	32.5	8.5	52 weeks	AF/AFL/VAs
AWARD-9, 2017	Dulaglutide	300	60.4	32.8	8.4	28 weeks	AF
AWARD-7, 2018	Dulaglutide	576	64.6	32.5	8.6	52 weeks	AF/SCD
AWARD-10, 2018	Dulaglutide	423	57.3	32.9	8.0	24 weeks	AF
Chen et al., 2018	Dulaglutide	735	52.8	25.9	8.0	26 weeks	VAs
REWIND, 2019	Dulaglutide	9892	66.2	32.3	7.3	282 weeks	AF/AFL/VAs/SCD
Heine et al., 2005	Exenatide	549	58.9	31.4	8.2	26 weeks	AF
Nauck et al., 2007	Exenatide	501	58.7	30.6	8.6	52 weeks	AF/AFL
NCT00701935, 2008	Exenatide	80	58.1	NA	NA	26 weeks	AF
EUREXA, 2012	Exenatide	1019	56.4	32.6	7.5	208 weeks	AF/AFL
DURATION-3, 2010	Exenatide	456	57.9	32.0	8.3	26 weeks	SCD
Inagaki et al., 2012	Exenatide	427	56.8	26.2	8.5	26 weeks	AF
Davies et al., 2013	Exenatide	216	58.5	33.7	8.4	26 weeks	VAs
LEAD-2, 2009	Liraglutide	1087	56.7	31.0	8.4	26 weeks	AF/AFL/VAs/SCD
LEAD-3 Mono, 2009	Liraglutide	746	53.0	33.1	8.3	104 weeks	AF
Pratley et al., 2010	Liraglutide	658	55.3	32.8	8.4	26 weeks	SCD
Charbonnel et al., 2012	Liraglutide	650	57.3	32.7	8.2	26 weeks	AF
MDI-Liraglutide, 2015	Liraglutide	124	63.7	33.7	9.0	24 weeks	AF
SCALE Obesity and Prediabetes, 2015	Liraglutide	3723	45.1	38.3	5.6	56 weeks	AF/AFL/VAs/SCD
Vanderheiden et al., 2016	Liraglutide	71	54.2	41.2	NA	26 weeks	AF
LEADER, 2016	Liraglutide	9340	64.3	32.5	8.7	198 weeks	AF/AFL/VAs/SCD
Zang et al., 2016	Liraglutide	367	51.5	27.2	8.1	26 weeks	AF
SCALE Insulin, 2020	Liraglutide	392	56.8	35.9	8.0	56 weeks	AF/AFL
GETGOAL-M, 2013	Lixisenatide	680	54.7	33.0	8.1	24 weeks	AF/VAs
GETGOAL-L, 2013	Lixisenatide	495	57.2	32.1	8.4	24 weeks	AF/AFL
GETGOAL-F1, 2014	Lixisenatide	482	56.1	32.5	8.0	24 weeks	AF
ELIXA, 2015	Lixisenatide	6063	60.3	30.2	7.7	107 weeks	AF/AFL/VAs/SCD
GetGoal-Duo-2, 2016	Lixisenatide	893	59.8	32.2	7.8	26 weeks	AF
PIONEER 2, 2019	Oral semaglutide	819	58.0	32.8	8.1	52 weeks	AF/VAs
PIONEER 3, 2019	Oral semaglutide	1861	58.0	32.5	8.3	78 weeks	AF/AFL/SCD
PIONEER 5, 2019	Oral semaglutide	324	70.0	32.4	8.0	26 weeks	AF
PIONEER 6, 2019	Oral semaglutide	3182	66.0	32.3	8.2	68 weeks	AF/AFL/VAs/SCD
PIONEER 8, 2019	Oral semaglutide	730	61.0	31.0	8.2	52 weeks	AFL/VAs
SUSTAIN 6, 2016	Sc semaglutide	3297	64.6	32.8	8.7	104 weeks	AF/AFL/VAs/SCD
SUSTAIN 2, 2017	Sc semaglutide	1225	55.1	32.5	8.1	56 weeks	AF
SUSTAIN 4, 2017	Sc semaglutide	1082	56.5	33.0	8.2	30 weeks	AF
Kaku et al., 2018	Sc semaglutide	600	58.5	26.4	8.1	56 weeks	AF
SUSTAIN China, 2021	Sc semaglutide	867	53.1	28.0	8.1	30 weeks	AF/VAs
STEP 1, 2021	Sc semaglutide	1961	46.0	37.8	5.7	68 weeks	AF
STEP 2, 2021	Sc semaglutide	1207	55.0	35.7	8.1	68 weeks	AF
STEP 4, 2021	Sc semaglutide	803	46.0	34.1	5.4	68 weeks	AF
STEP 6, 2022	Sc semaglutide	400	51.0	31.9	6.	68 weeks	AF
SUSTAIN 11, 2022	Sc semaglutide	1738	61.2	31.5	8.6	52 weeks	AF/AFL/SCD

*AF* atrial fibrillation, *AFL* atrial flutter, *BMI* body mass index, *GLP-1 RAs* glucagon-like peptide 1 receptor agonists, *NA* not available, *Sc* subcutaneous injection, *SCD* sudden cardiac death, *VAs* ventricular arrhythmias

According to the revised RoB2 tool for assessing the quality of RCTs, there was no study with a high risk of bias. Most studies had a low risk or some bias issues in the five domains assessed (Additional file 1: Fig. S1).

### Atrial fibrillation

**Fig. 2:**
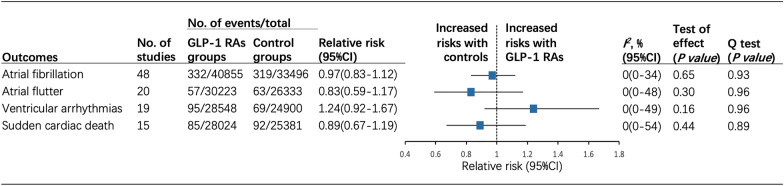
Risk of cardiac arrhythmias in patients randomized to GLP-1 RAs treatment compared with controls across all trials. *GLP-1 RAs glucagon-like peptide 1 receptor agonists*, *CI* confidence interval

**Fig. 3:**
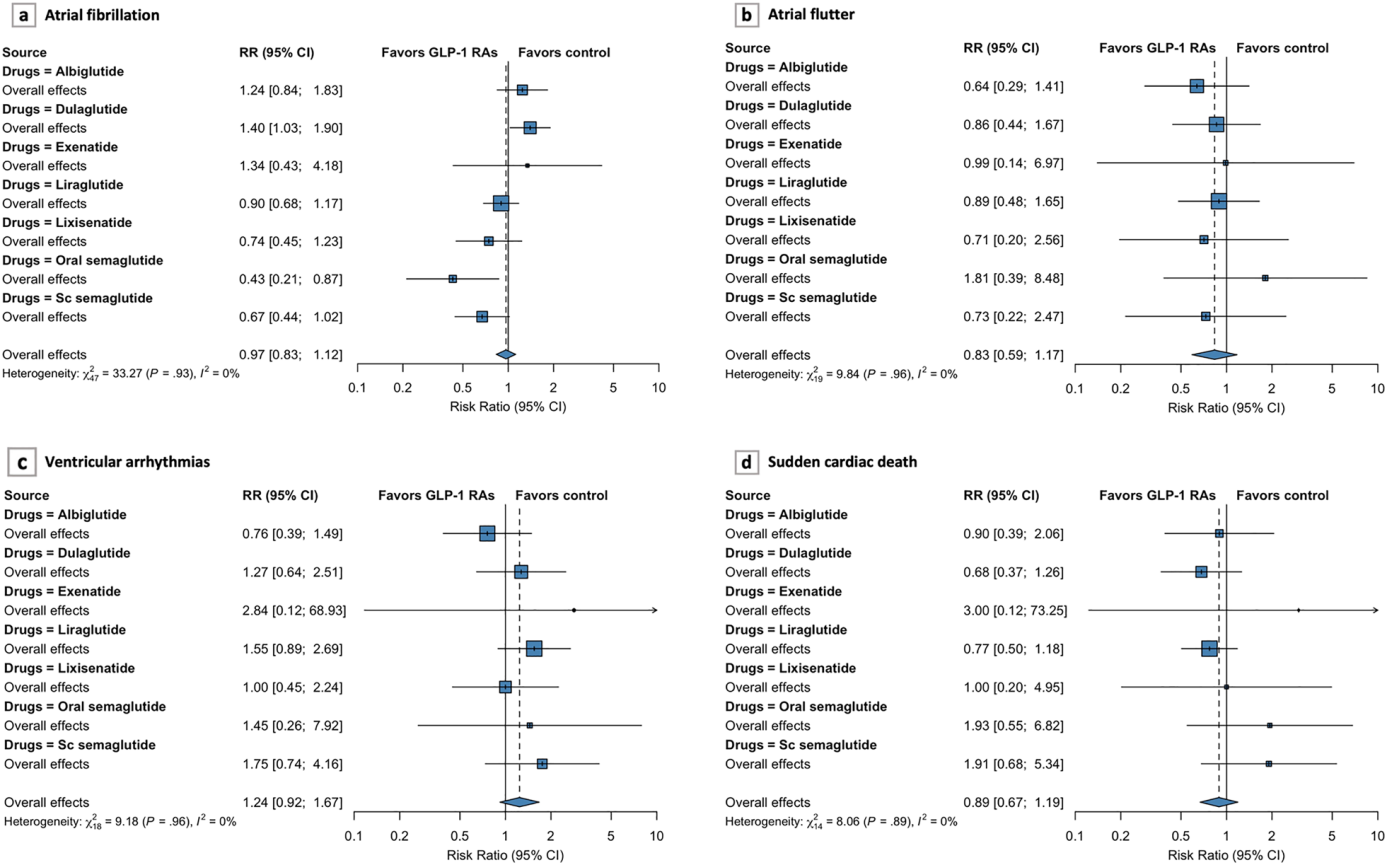
Forest plots of primary analysis for each outcome. **a** atrial fibrillation, **b** atrial flutter, **c** ventricular arrhythmias, **d** sudden cardiac death. Overall effects are calculated using the fixed‑effect model

48 trials had reported a total of 651 events of AF as SAEs in 74,351 participants (332 events in GLP-1 RAs group and 319 events in control group) (Fig. 2). Pooled data from all these trials indicated that GLP-1 RAs therapy had no significant association with the risk of incident AF (RR 0.97, 95% CI 0.83–1.12; *P* = 0.65) (Additional file 1: Fig. S2.1). Regarding the association of AF risk and different GLP-1 RAs agents, dulaglutide displayed an increasing trend toward incident AF (RR 1.40, 95% CI 1.03–1.90; *P* = 0.03), while oral semaglutide displayed an inverse trend (RR 0.43, 95% CI 0.21–0.87; *P* = 0.02) (Fig. 3a). Other GLP-1 RAs agents including albiglutide, exenatide, liraglutide, lixisenatide, and sc semaglutide had no significant effect on the risk of AF. Overall, the cumulative incidence of AF was low, averaged at 39 per 10,000 patient-years in GLP-1 RAs treatment. In the subgroup analyses, the incidence of AF did not differ between the GLP-1 RAs and control groups according to different treatment dosage, follow-up duration, baseline BMI, and study designs, with no intergroup heterogeneity (*P* = 0.72, *P* = 0.59, *P* = 0.08, and *P* = 0.70, respectively) (Table 2 and Additional file 1: Fig. S3).

**Table 2:** Results of subgroup analyses

Subgroups	No. of patients	No. of studies	RR (95% CI)	^a^*P*_effect_	Heterogeneity		^b^*P*_hetero_
					*I*^2^, %	*P* value	
Atrial fibrillation							
Dose^c^							0.72
High	42,809	26	0.97 (0.81–1.17)	0.77	0	0.55	
Low	22,224	19	1.04 (0.77–1.40)	0.81	0	0.94	
Follow-up duration							0.59
≤ 52 weeks	25,271	32	0.89 (0.62–1.26)	0.50	0	0.99	
> 52 weeks	49,080	16	0.99 (0.84–1.16)	0.86	13	0.30	
Baseline BMI^d^							0.08
High	34,143	27	0.84 (0.67–1.04)	0.11	0	0.99	
Low	39,562	19	1.10 (0.89–1.35)	0.38	0	0.55	
Study designs							0.70
CVOT^e^	41,206	6	0.93 (0.84–1.17)	0.60	58	0.04	
Non-CVOT	33,145	42	0.84 (0.56–1.27)	0.41	0	1.00	
Atrial flutter							
Dose							0.34
High	33,626	10	1.04 (0.68–1.58)	0.86	0	0.79	
Low	14,949	7	0.70 (0.35–1.40)	0.31	0	0.63	
Follow-up duration							0.19
≤ 52 weeks	8355	10	1.52 (0.58–3.97)	0.40	0	1.00	
> 52 weeks	48,201	10	0.76 (0.52–1.10)	0.14	0	0.66	
Baseline BMI							0.90
High	21,261	8	0.86 (0.50–1.47)	0.58	0	0.79	
Low	35,295	12	0.82 (0.53–1.27)	0.37	0	0.88	
Study designs							0.46
CVOT	41,206	6	0.78 (0.53–1.15)	0.21	0	0.48	
Non-CVOT	15,350	14	1.08 (0.51–2.30)	0.84	0	0.98	
Ventricular arrhythmias							
Dose							0.04
High	32,205	11	1.63 (1.11–2.40)	0.01	0	0.96	
Low	16,142	9	0.83 (0.49–1.41)	0.49	0	0.91	
Follow-up duration							0.47
≤ 52 weeks	7712	11	0.93 (0.41–2.12)	0.86	0	0.93	
> 52 weeks	45,736	8	1.29 (0.94–1.78)	0.12	0	0.72	
Baseline BMI							0.13
High	20,459	9	1.60 (1.04–2.48)	0.03	0	0.96	
Low	32,423	9	1.00 (0.66–1.52)	1.00	0	0.89	
Study designs							0.63
CVOT	41,206	6	1.28 (0.92–1.77)	0.14	0	0.50	
Non-CVOT	12,242	13	1.04 (0.48–2.24)	0.92	0	0.97	
Sudden cardiac death							
Dose							0.39
High	33,360	10	0.84 (0.61–1.17)	0.31	0	0.94	
Low	16,314	8	1.13 (0.63–2.04)	0.68	0	0.76	
Follow-up duration							0.65
≤ 52 weeks	5603	6	0.70 (0.24–2.05)	0.52	0	0.60	
> 52 weeks	47,802	9	0.91 (0.67–1.22)	0.52	0	0.84	
Baseline BMI							0.80
High	20,467	7	0.86 (0.59–1.26)	0.45	0	0.58	
Low	32,938	8	0.93 (0.60–1.44)	0.75	0	0.86	
Study designs							0.90
CVOT	41,206	6	0.90 (0.66–1.22)	0.49	0	0.63	
Non-CVOT	12,199	9	0.84 (0.35–2.04)	0.71	0	0.80	

*BMI* body mass index, *CVOT* cardiovascular outcome trial

^a^*P*_effect_, *P*-value for test effect

^b^*P*_hetero_, *P*-value for between-subgroup heterogeneity

^C^Only studies of albiglutide, dulaglutide, liraglutide, and semaglutide (oral and subcutaneous) had different treatment doses (a single trial may have one or more dose sizes)

^d^The cut-off point was 32.38 kg/m^2^ (mean baseline BMI in all included trials except 2 trials without reporting data on baseline BMI)

^e^Random effects model were used to calculate the estimates because of the high heterogeneity

### Atrial flutter

A total of 57 patients treated with GLP-1 RAs experienced AFL (n = 30,223), whereas 63 individuals in the control group (n = 26,333). The cumulative incidence of AFL with GLP-1 RAs was 8 per 10,000 patient-years. GLP-1 RAs use did not significantly increase the incidence of AFL (RR 0.83, 95% CI 0.59–1.17; *P* = 0.96) compared to controls (Additional file 1: Fig. S2.2). The same trend was obtained in the analysis of different groups of GLP-1 RAs receivers (Fig. 3b). There was no significant heterogeneity among all included trials on AFL (*I*^2^ = 0%, *P* = 0.96). Similar nonsignificant association and absence of intergroup heterogeneity were also found in the subgroup analyses (Table 2 and Additional file 1: Fig. S4).

### Ventricular arrhythmias

164 events of VAs were reported in 19 trials, 95 of which occurred in the GLP-1 RAs group (n = 28,548). The cumulative incidence of VAs with GLP-1 RAs was nearly 14 per 10,000 patient-years. The aggregated data showed that GLP-1 RAs therapy did not increase the risk of VAs compared to the control (RR 1.24, 95% CI 0.92–1.67; *P* = 0.16) (Additional file 1: Fig. S2.3). When focusing on the treatment dose, an increased incidence of VAs was identified in the high-dose GLP-1 RAs group (RR 1.63, 95% CI 1.11–2.40; *P* = 0.01) but not in the low-dose group (RR 0.83, 95% CI 0.49–1.41; *P* = 0.49) with some intergroup heterogeneity (*P* = 0.04) (Additional file 1: Fig. S5.2). Subgroup analysis by baseline BMI indicated that an increase in VAs incidence was pronounced in those with higher baseline BMI (RR 1.60, 95% CI 1.04–2.48; *P* = 0.03) but not in those with low BMI (RR 1.00, 95% CI 0.66–1.52; *P* = 1.00), with no intergroup heterogeneity (*P* = 0.13) (Additional file 1: Fig. S5.4). Both follow-up duration and study designs were not significantly linked with the incidence of VAs, and had no between-subgroup heterogeneity (*P* = 0.47, and *P* = 0.63, respectively) (Table 2). No significant heterogeneity was found across trials on VAs (*I*^2^ = 0%, *P* = 0.96).

### Sudden cardiac death

A total of 85 SCD events and 8 per 10,000 patient-years SCD incidence in GLP-1 RAs group were reported. The RRs for SCD ranged from 0.17 to 3.00. The risk of incident SCD was not significantly different between GLP-1 RAs and control (RR 0.89; 95% CI 0.67–1.19; *P* = 0.51) (Additional file 1: Fig. S2.4). No significant heterogeneity was identified across trials on SCD (*I*^2^ = 0%, *P* = 0.89). In subgroup analyses, GLP-1 RAs were not significantly associated with the risk of SCD when grouped according to different treatment dosage, follow-up duration, baseline BMI, and study designs. Likewise, there is no intergroup heterogeneity (*P* = 0.39, *P* = 0.65, *P* = 0.80, and *P* = 0.90, respectively) (Table 2 and Additional file 1: Fig. S6).

### Sensitivity analyses and publication bias

Sensitivity analyses for each endpoint were conducted in turn. After the iterative omission of each trial, the pooled results for each outcome would not change (Additional file 1: Fig. S7). Considering the variation in the sample size across studies, we performed further sensitivity analyses in the aforementioned subgroups with significant results. After removing the REWIND trial and the POINEER-6 trial respectively, the pooling effect of dulaglutide and oral semaglutide on the risk of AF would lose statistical significance (Additional file 1: Fig. S7.5–6). When we removed the SCALE trial and the SUSTAIIN-6 trial, the previously observed statistically significant association between GLP-1 RAs and VAs in the higher-BMI and higher-doses subgroup would diminish. (Additional file 1: Fig. S7.7–8). Funnel plots for AF, AFL, VAs, and SCD displayed symmetry (Additional file 1: Fig. S8). Egger’s test for each outcome did not reveal significant asymmetry (*P* = 0.23, *P* = 0.38, *P* = 0.49, *P* = 0.55, respectively).

## Discussion

In this systematic review and meta-analysis of 56 RCTs and 79,720 patients with T2DM or obesity, GLP-1 RAs therapy was not associated with an overall higher risk of incident AF, AFL, VAs, and SCD. Apart from VAs, the point estimates in relation to other conditions, all suggested potential benefits of GLP-1 RAs compared to the placebo or active group. Additionally, we observed a potentially protective effect of oral semaglutide on AF but an inverse trend of dulaglutide. When considering VAs, GLP-1 RAs might be associated with an increased risk of VAs in higher dosage and in those with higher baseline BMI.

To our knowledge, this is the largest systematic review and meta-analysis that comprehensively evaluated the relationship between GLP-1 RAs use and arrhythmia outcomes. In accordance with several previous systematic reviews [17–19], our study found that GLP-1 RAs did not increase the risk of cardiac arrhythmias and cemented its cardiovascular safety. A recent network meta-analysis demonstrated differently that GLP-1 RAs might significantly reduce the risk of AF/AFL in diabetic patients compared with other hypoglycemic drugs [20]. Nevertheless, these existing reviews were limited to a few included studies with restrictions on populations and did not include several important studies that have been published recently [21–24].

AF was the most reported arrhythmia event in the present study, consistent with its high prevalence [25]. The incidence of AF in our study was close to a previous cohort study (39 per 10,000 patient-years vs 32 per 10,000 patient-years) [26]. Although our meta-analysis failed to identify a significant association between GLP-1 RAs and AF, it is noted that oral semaglutide had a protective effect on incident AF. Interestingly, subcutaneous semaglutide also seemed to be associated with a small, albeit marginally significant reduction in AF events. These results support the findings of the prior meta-analysis [20], also demonstrating similar efficacy of oral and subcutaneous administration. A slight trend toward an increased risk of AF was observed with dulaglutide, whereas the trend was not robust owing to the overweighting of the REWIND trial. We did not observe a significant association with AF in other GLP-1 RAs agents, suggesting a possible drug-specific effect. Larger studies evaluating the effects of GLP-1 RAs on AF are required to confirm the association.

According to our findings, the incidence of AFL appeared to be lower in the GLP-1RAs group than in the control group, but the difference did not reach statistical significance. In fact, AFL and AF share similar clinical presentations and consequences. AF/AFL has been firmly established to increase the risk of stroke, approximately 13-26% of ischemic strokes are attributed to AF/AFL [27]. A recent cohort study of patients with T2DM observed that GLP-1 RAs treatment could exert a protective effect against ischemic stroke [28]. A speculation was proposed that a reduction in AF/AFL might play an important role in this observation. Owing to the relatively low incidence of AFL, future trials could specify AF and AFL as a composite outcome. And the conjoint analysis of AF/AFL would provide more robust results while obviating possible publication bias for AFL.

The current study found that both higher dose of GLP-1RAs and higher baseline BMI were linked with a nearly 60% higher risk of incident VAs. In practice, GLP-1 RAs are often given in higher doses for weight loss than for glycaemic control. The US Food and Drug Administration (FDA) has approved liraglutide (3.0 mg, daily) and subcutaneous semaglutide (2.4 mg, weekly) for weight management, which are irregular doses for diabetic control. Up-titration of GLP-1 RAs, on the other hand, may be recommended for T2DM patients who do not reach glycemic control targets with lower dosages. Considering the increasing risk of VAs, we should be more careful when treating obese subjects or giving higher dosage to T2DM patients.

The relationship between GLP-1 RAs and SCD has been less well reported, and the current meta-analysis is the first to summarize the SCD events in GLP-1 RAs trials. In our study, GLP-1 RAs treatment appeared to lack of significant effect on SCD events. Further subgroup analyses also supported this finding. In T2DM patients, SCD is partly mediated by the increased presence of coronary heart disease, which facilitates the occurrence of malignant VAs [29], such as ventricular fibrillation and ventricular flutter. However, both VAs and SCD events are difficult to detect since they tend to have immediate onsets and short duration, which may lead to under-reporting. Considering the serious hazard, future trials of GLP-1 RAs should fully detect and report VAs and SCD events.

The pharmacological and physiological mechanisms linking GLP-1RAs and arrhythmias remain unknown. GLP-1 RAs act by activating GLP-1 receptors (GLP-1R) widely distributed outside the pancreas [30]. GLP-1R has also been expressed in the cardiovascular system, within the heart and blood vessels [31, 32]. Some clinical and preclinical studies have demonstrated that the contribution to cardiovascular protection of GLP-1 RAs is mainly ascribed to its modest reduction of blood pressure [33], correction of dyslipidemia [34], improvement of microvascular function [35, 36], and anti-oxidization or anti-inflammation [1, 37]. Since the distributions of GLP-1R in sino-atrial node and ventricular cardiomyocytes [31, 38], a previous study indicated that the direct activations of GLP-1R effectively opposed the effects of β-adrenoceptor stimulation on cardiac ventricular excitability and might be responsible for HR augmentation or arrhythmias [39]. An alternative explanation is that GLP-1RAs may act on the autonomic nervous system. An observational study with 60 T2DM patients showed that liraglutide increased cardiac sympathetic nervous system activity compared to baseline measurements [40]. However, in a study that enrolled 28 T2DM patients, the cardiac sympatho-vagal balance (assessed by HR variability) was not affected after 3 or 6 months of exenatide treatment, despite an increase in HR [41]. Thus, the underlying mechanism by which GLP-1 RAs influence the incidence and development of arrhythmias requires further exploration.

It is well-recognized that both diabetes and obesity are associated with AF and other cardiac arrhythmias, which may in turn increase adverse cardiovascular events and mortality in diabetic and obese individuals [42–44]. Therefore, it is critically important to reduce the risk of cardiac arrhythmias in T2DM and obese patients. Our study showed that GLP-1 RAs might produce antiarrhythmic or arrhythmogenic effects under certain circumstances. However, previous trials mainly reported arrhythmic events as SAEs, further large-scale controlled studies should pre-specify arrhythmias as endpoints to confirm our findings. Additionally, it is possible that GLP-1 RAs are drug-specific and have individual variability in different populations. More evidence is required to assist clinicians in the selection of suitable GLP-1 RAs agents and optimal populations based on the risk classification of arrhythmias. Studies are also needed to help determine the pathophysiological mechanisms by which GLP-1 RAs increase heart rate and generate potential antiarrhythmic or arrhythmogenic effects.

The present systematic review and meta-analysis was strengthened by incorporating recently published trials and larger populations, and by reporting more arrhythmia outcomes. Moreover, we conducted extended and detailed subgroup analyses to investigate the potential risk factors affecting the development of arrhythmias. These made our results more convincing and robust. However, there are several limitations in our study. Firstly, the incidence of arrhythmias was relatively low and even absent from some included studies, leading to wide confidence intervals. Hence, these results might be influenced by low power. Secondly, we included trials involving both diabetic and obese patients, and this population heterogeneity might influence the pooled results. However, diabetes and obesity are both metabolic disorders, and there is a growing recognition that obesity, diabetes, and arrhythmias (especially AF) are closely intertwined epidemics. In addition to T2DM, another key indication for GLP-1 RAs is obesity. Obese patients tend to receive higher-dosage GLP-1 RAs and prolonged treatment duration. To evaluate the cardiovascular safety of GLP-1 RAs and fully understand the utilization of this drug, it is necessary to include these populations. Thirdly, there were some changes mainly related to outcomes in the research protocol registered on the PROSPERO website. To achieve a more comprehensive assessment of arrhythmia outcomes, we added incident AFL as an outcome. Additionally, considering the complexity of the etiologic composition of SCD, we specified VAs and SCD as independent outcomes. We made these alterations before we conducted data extraction and analysis. We do not think that these alterations would change our conclusions; instead, we believe that these alterations would enhance the rigor of our study. Finally, although this was a meta-analysis of numerous RCTs, the results of which were less sensitive and convincing than the analysis of individual-level data.

## Conclusions

GLP-1 RAs therapy was overall not significantly associated with incident arrhythmias in diabetic and obese patients, suggesting an assuring cardiovascular safety profile. Oral semaglutide might be associated with a lower risk of incident AF while dulaglutide demonstrated opposite effect. Of note, given the increased risk of VAs, physicians should be more careful when treating obese subjects or prescribing high dosage. Furthermore, the potential antiarrhythmic or arrhythmogenic effect of GLP-1 RAs may be drug-specific and require further investigation.

## Supplementary Information

**Additional file 1: Method S1.** Data sources and search strategies. **Table S1.** Eligible criteria for included studies. **Table S2.** Baseline characteristics of included studies and participants. **Figure S1.** Methodological quality assessment of included studies. **Figure S2.** Risks of cardiac arrhythmias in patients with GLP-1 RAs treatments compared with control groups. **Figure S3.** Subgroup analyses on the association of GLP-1 RAs use with incident atrial fibrillation. **Figure S4.** Subgroup analyses on the association of GLP-1 RAs use with the incidence of atrial flutter. **Figure S5.** Subgroup analyses on the association of GLP-1 RAs use with the incidence of ventricular arrhythmias. **Figure S6.** Subgroup analyses on the association of GLP-1 RAs use with the incidence of sudden cardiac death. **Figure S7.** Sensitivity analyses by omitting each trial one by one of all included studies. **Figure S8.** Funnel plots for each outcome. **References S1.**

## Data Availability

All relevant data generated or analyzed in our study are available in the main text and the additional file 1.

## Abbreviations

GLP-1 RAs: Glucagon-like peptide-1 receptor agonists
GLP-1: Glucagon-like peptide-1
HR: Heart rate
AF: Atrial fibrillation
AFL: Atrial flutter
VAs: Ventricular arrhythmias
SCD: Sudden cardiac death
T2DM: Type 2 diabetes mellitus
BMI: Body mass index
HbA1c: Glycated hemoglobin
MACE: Major adverse cardiovascular event
RR: Relative risk
CI: Confidence interval
RCT: Randomized controlled trial
CVOT: Cardiovascular outcome trial
SAE: Severe adverse effect
Sc: Subcutaneous injection
PRISMA: Preferred Reporting Items for Systematic Reviews and Meta-Analyses
FDA: Food and Drug Administration
