# Supplementary material for: Association of glucagon-like peptide-1 receptor agonists with cardiac arrhythmias in patients with type 2 diabetes or obesity: a systematic review and meta-analysis of randomized controlled trials
Source: Diabetol Metab Syndr. 2022 Dec 26;14:195. doi: 10.1186/s13098-022-00970-2 (PMC9791739; doi:10.1186/s13098-022-00970-2)
Supplement: Supplementary file 1 — Additional file 1: Method S1. Data sources and search strategies. Table S1. Eligible criteria for included studies. Table S2. Baseline characteristics of included studies and participants. Figure S1. Methodological quality assessment of included studies. Figure S2. Risks of cardiac arrhythmias in patients with GLP-1 RAs treatments compared with control groups. Figure S3. Subgroup analyses on the association of GLP-1 RAs use with incident atrial fibrillation. Figure S4. Subgroup analyses on the association of GLP-1 RAs use with the incidence of atrial flutter. Figure S5. Subgroup analyses on the association of GLP-1 RAs use with the incidence of ventricular arrhythmias. Figure S6. Subgroup analyses on the association of GLP-1 RAs use with the incidence of sudden cardiac death. Figure S7. Sensitivity analyses by omitting each trial one by one of all included studies. Figure S8. Funnel plots for each outcome. References S1. [file 13098_2022_970_MOESM1_ESM.pdf]

Supplementary Context

An additional file of the manuscript entitled "Association of glucagon-like peptide-1 receptor agonists with cardiac arrhythmias in patients with type 2 diabetes or obesity: a systematic review and meta-analysis of randomized controlled trials"

Method S1. Data sources and search strategies

Table S1. Eligible criteria for included studies

Table S2. Baseline characteristics of included studies and participants

Figure S1. Methodological quality assessment of included studies

Figure S2. Risks of cardiac arrhythmias in patients with GLP-1 RAs treatments compared with control groups

Figure S3. Subgroup analyses on the association of GLP-1 RAs use with incident atrial fibrillation

Figure S4. Subgroup analyses on the association of GLP-1 RAs use with the incidence of atrial flutter

Figure S5. Subgroup analyses on the association of GLP-1 RAs use with the incidence of ventricular arrhythmias

Figure S6. Subgroup analyses on the association of GLP-1 RAs use with the incidence of sudden cardiac death

Figure S7. Sensitivity analyses by omitting each trial one by one of all included studies

Figure S8. Funnel plots for each outcome

References S1

Method S1. Data Sources and Search strategies

Example of a search strategy run in Pubmed

#1
TS= ((((((((((("glucagon like peptide 1 receptor agonist"[Title/Abstract]) OR ("glp 1 agonist"[Title/Abstract])) OR ("glp 1 receptor agonist"[Title/Abstract])) OR ("glucagon like peptide 1 agonist"[Title/Abstract])) OR ("long acting glp 1 agonist"[Title/Abstract])) OR ("long acting glp 1 receptor agonist"[Title/Abstract])) OR ("long acting glucagon like peptide 1 receptor agonist"[Title/Abstract])) OR (dulaglutide[Title/Abstract])) OR (liraglutide[Title/Abstract])) OR (exenatide[Title/Abstract])) OR (albiglutide[Title/Abstract])) OR (semaglutide[Title/Abstract])) OR (lixisenatide[Title/Abstract])

#2
TS= (((((("random allocation") OR ("randomized controlled trial")) OR ("controlled clinical trial")) OR ("clinical trial")) OR (randomi?ed)) OR (randomly)) OR (randomization)

#3
TS=("1948/01/01"[Date - Publication] : "2022/05/25"[Date - Publication])

#4
#1 AND #2 AND #3

Table S1. Eligible criteria for included studies

Inclusion criteria	Exclusion criteria
(1) RCTs with at least one arm including GLP-1 RAs medications.<br>(2) Presence of a control group (either placebo-controlled or active-controlled).<br>(3) Adult patients older than 18 years with diagnosed T2DM/prediabetes, obesity/overweight, or both.<br>(4) The duration of treatments was at least 24 weeks.	(1) Review, comment, guideline, conference abstract, basic studies, case report, non-RCT clinical studies, or other irrelevant studies.<br>(2) Post hoc analysis or exploratory analysis of existing RCTs.<br>(3) Participants with GLP-1 RAs treatments before primary trials.<br>(4) Participants with T1DM or other diseases (e.g., AD, PD, or NAFLD) besides T2DM and obesity.<br>(5) Compound preparations of GLP-1 RAs and other drugs(e.g., IDegLira).<br>(6) Comparisons between different GLP-1 RAs individuals.<br>(7) Data is not available or fails to report the events of AF/AFL, VAs, or SCD.<br>(8) Excluding trials with zero events in both the GLP-1 RAs group and control group.

Abbreviations: GLP-1 RAs, glucagon-like peptide 1 receptor agonists; T2DM, Type 2 diabetes mellitus; RCT, randomized controlled trial; AD, Alzheimer's disease; PD, Parkinson's disease; NAFLD, non-alcoholic fatty liver disease; AF, atrial fibrillation; AFL, atrial flutter; VAs, ventricular arrhythmias; SCD, sudden cardiac death.

Table S2. Baseline characteristics of included studies and participants

Trials, year	Registration number	Inclusion criteria	Population size	Males, n(%)	Age (years)	BMI (kg/m^2)	HbA1c	Follow-up duration	Interventions	Controls	Outcomes of interest
HARMONY 1, 2014[1]	NCT00849056	T2DM with pioglitazone	301	180(59.8%)	55.0	34.1	8.1%	52 weeks	Albiglutide 30mg, weekly	Placebo	AF
HARMONY 3, 2014[2]	NCT00838903	T2DM with metformin	1012	482(47.6%)	54.5	32.7	8.1%	104 weeks	Albiglutide 30mg, weekly	Sitagliptin, glimepiride, placebo	SCD
HARMONY 4, 2014[3]	NCT00838916	T2DM with metformin	745	418(56.1%)	55.5	33.1	8.3%	52 weeks	Albiglutide 30mg, weekly	Insulin glargine	AF/AFL/VAs
HARMONY 8, 2014[4]	NCT01098539	T2DM with renal impairment	495	266(53.7%)	63.3	30.4	8.2%	52 weeks	Albiglutide 30mg, weekly	Sitagliptin	AF/AFL/VAs
Rosenstock et al, 2014[5]	NCT00976391	T2DM with insulin	566	268(47.3%)	55.6	NA	8.5%	26 weeks	Albiglutide 30mg, weekly	Insulin lispro	AF/VAs
HARMONY 5, 2015[6]	NCT00839527	T2DM with OAD	663	353(53.2%)	55.2	32.2	8.2%	52 weeks	Albiglutide 30mg, weekly	Pioglitazone, placebo	AF
HARMONY 2, 2016[7]	NCT00849017	T2DM with diet and exercise control	301	166(55.1%)	52.9	33.9	8.2%	52 weeks	Albiglutide 30mg/50mg, weekly	Placebo	AF
Harmony Outcomes, 2018[8]	NCT02465515	T2DM with CVD	9432	6569(69.4%)	64.1	32.3	8.7%	78 weeks	Albiglutide 30/50mg, weekly	Placebo	AF/AFL/VAs/SCD
Rosenstock et al, 2020[9]	NCT02229227	T2DM with insulin	813	372(45.7%)	58.1	32.1	7.7%	26 weeks	Albiglutide 30mg, weekly	Insulin lispro	AF/AFL
Ferdinand et al, 2014[10]	NCT01149421	T2DM with OAD	755	392(51.9%)	56.5	33.0	7.9%	26 weeks	Dulaglutide 0.75mg/1.5mg, weekly	Placebo	VAs
AWARD-3, 2014[11]	NCT01126580	T2DM with OAD	807	353(43.7%)	55.6	33.3	7.6%	52 weeks	Dulaglutide 0.75mg/1.5mg, weekly	metformin	AF
AWARD-5, 2014[12]	NCT00734474	T2DM with metformin	1098	559(46.5%)	54.0	31.3	8.1%	52 weeks	Dulaglutide 0.75mg/1.5mg, weekly	Sitagliptin, placebo	AF/SCD
AWARD-2, 2015[13]	NCT01075282	T2DM with OAD	807	414(51.3%)	56.6	31.6	8.1%	78 weeks	Dulaglutide 0.75mg/1.5mg, weekly	Insulin glargine	VAs
AWARD-4, 2015[14]	NCT01191268	T2DM with insulin	884	473(53.5%)	59.4	32.5	8.5%	52 weeks	Dulaglutide 0.75mg/1.5mg, weekly	Insulin glargine	AF/AFL/VAs

Trials, year	Registration number	Inclusion criteria	Population size	Males, n(%)	Age (years)	BMI (kg/m^2)	HbA1c	Follow-up duration	Interventions	Controls	Outcomes of interest
AWARD-9, 2017[15]	NCT02152371	T2DM with insulin glargine	300	173(57.7%)	60.4	32.8	8.4%	28 weeks	Dulaglutide 1.5mg, weekly	Placebo	AF
AWARD-7, 2018[16]	NCT01621178	T2DM with CKD	576	301(52.2%)	64.6	32.5	8.6%	52 weeks	Dulaglutide 0.75mg/1.5mg, weekly	Insulin glargine	AF/SCD
AWARD-10, 2018[17]	NCT02597049	T2DM with SGLT2i	423	212(50.1%)	57.3	32.9	8.0%	24 weeks	Dulaglutide 0.75mg/1.5mg, weekly	Placebo	AF
Chen et al, 2018[18]	NCT01644500	T2DM with OAD	735	391(54.3%)	52.8	25.9	8.0%	26 weeks	Dulaglutide 0.75mg/1.5mg, weekly	Glimepiride	VAs
REWIND, 2019[19]	NCT01394952	T2DM with previous CVD or CVD risk	9892	5312(53.7%)	66.2	32.3	7.3%	282 weeks	Dulaglutide 1.5mg, weekly	Placebo	AF/AFL/VAs/SCD
Heine et al, 2005[20]	NCT00082381	T2DM with OAD	549	306(55.7%)	58.9	31.4	8.2%	26 weeks	Exenatide 10μg, twice daily	Insulin glargine	AF
Nauck et al, 2007[21]	NCT00082407	T2DM with OAD	501	244(48.7%)	58.7	30.6	8.6%	52 weeks	Exenatide 10μg, twice daily	Biphasic Insulin	AF/AFL
NCT00701935, 2008[22]	NCT00701935	T2DM with metformin	80	42(52.5%)	58.1	NA	NA	26 weeks	Exenatide 10μg, twice daily	Placebo	AF
EUREXA, 2012[23]	NCT00359762	T2DM with metformin	1019	524(53.6%)	56.4	32.6	7.5%	208 weeks	Exenatide 10μg, twice daily	Glimepiride	AF/AFL
DURATION-3, 2010[24]	NCT00641056	T2DM with OAD	456	243(53.3%)	57.9	32.0	8.3%	26 weeks	Exenatide 2mg, weekly	Insulin glargine	SCD
Inagaki et al, 2012[25]	NCT00935532	T2DM with OAD	427	290(67.9%)	56.8	26.2	8.5%	26 weeks	Exenatide 2mg, weekly	Insulin glargine	AF
Davies et al, 2013[26]	NCT01003184	T2DM with metformin	216	143(66.2%)	58.5	33.7	8.4%	26 weeks	Exenatide 2mg, weekly	Insulin detemir	VAs
LEAD-2, 2009[27]	NCT00318461	T2DM with metformin	1087	633(58.2%)	56.7	31.0	8.4%	26 weeks	Liraglutide 0.6mg/1.2mg/1.8mg, daily	Glimepiride, placebo	AF/AFL/VAs/SCD
LEAD-3 Mono, 2009[28]	NTC00294723	T2DM with OAD	746	371(49.7%)	53.0	33.1	8.3%	104 weeks	Liraglutide 1.2mg/1.8mg, daily	Glimepiride	AF
Pratley et al, 2010[29]	NCT00700817	T2DM with metformin	658	352(52.9%)	55.3	32.8	8.4%	26 weeks	Liraglutide 1.2mg/1.8mg, daily	Sitagliptin	SCD

Trials, year	Registration number	Inclusion criteria	Population size	Males, n(%)	Age (years)	BMI (kg/m^2)	HbA1c	Follow-up duration	Interventions	Controls	Outcomes of interest
Charbonnel et al, 2013[30]	NCT01296412	T2DM with metformin	650	358(54.8%)	57.3	32.7	8.2%	26 weeks	Liraglutide 0.6mg/1.2mg, daily	Sitagliptin	AF
MDI-Liraglutide, 2015[31]	EudraCT 2012-001941-42	T2DM with insulin, BMI 27.5-45 kg/m^2	124	80(64.5%)	63.7	33.7	9.0%	24 weeks	Liraglutide 1.8mg, daily	Placebo	AF
SCALE Obesity and Prediabetes, 2015[32]	NCT01272219	Obesity with BMI≥30kg/m^2, or BMI≥27kg/m^2 with dyslipidemia	3723	803(21.5%)	45.1	38.3	5.6%	56 weeks	Liraglutide 3mg, daily	Placebo	AF/AFL/VAs/SCD
Vanderheiden et al, 2016[33]	NCT01505673	T2DM with insulin	71	26(36.6%)	54.2	41.2	NA	26 weeks	Liraglutide 1.8mg, daily	Placebo	AF
LEADER, 2016[34]	NCT01179048	T2DM with high CVD risk	9340	6003(64.3%)	64.3	32.5	8.7%	198 weeks	Liraglutide 1.8mg, daily	placebo	AF/AFL/VAs/SCD
Zang et al, 2016[35]	NCT02008682	T2DM with metformin	367	219(59.7%)	51.5	27.2	8.1%	26 weeks	Liraglutide 1.8mg, daily	Sitagliptin	AF
SCALE Insulin, 2020[36]	NCT02963922	Overweight or obesity; or T2DM with insulin	392	189(47.7%)	56.8	35.9	8.0%	56 weeks	Liraglutide 3mg, daily	Placebo	AF/AFL
GETGOAL-M, 2013[37]	NCT00712673	T2DM with metformin	680	293(43.1%)	54.7	33.0	8.1%	24 weeks	Lixisenatide 20 ug, daily	Placebo	AF/VAs
GETGOAL-L, 2013[38]	NCT00715624	T2DM with insulin or OAD	495	228(46.1%)	57.2	32.1	8.4%	24 weeks	Lixisenatide 20 ug, daily	Placebo	AF/AFL
GETGOAL-F1, 2014[39]	NCT00763451	T2DM with metformin	482	215(44.6%)	56.1	32.5	8.0%	24 weeks	Lixisenatide 20 ug, daily	Placebo	AF
ELIXA, 2015[40]	NCT01147250	T2DM with a recent acute coronary event	6063	4207(69.3%)	60.3	30.2	7.7%	107 weeks	Lixisenatide 20 ug, daily	Placebo	AF/AFL/VAs/SCD
GetGoal-Duo-2, 2016[41]	NCT01768559	T2DM with overweight	893	405(45.3%)	59.8	32.2	7.8%	26 weeks	Lixisenatide 20 ug, daily	Insulin Glulisine	AF

Trials, year	Registration number	Inclusion criteria	Population size	Males, n(%)	Age (years)	BMI (kg/m^2)	HbA1c	Follow-up duration	Interventions	Controls	Outcomes of interest
PIONEER 2, 2019[42]	NCT02863328	T2DM with metformin	819	415(50.5%)	58.0	32.8	8.1%	52 weeks	Oral semaglutide 14mg, daily	Empagliflozin	AF/VAs
PIONEER 3, 2019[43]	NCT02607865	T2DM with metformin	1861	984(52.8%)	58.0	32.5	8.3%	78 weeks	Oral semaglutide 3/7/14mg, daily	Sitagliptin	AF/AFL/SCD
PIONEER 5, 2019[44]	NCT02827708	T2DM with moderate renal impairment	324	156(48.1%)	70.0	32.4	8.0%	26 weeks	Oral semaglutide 14mg, daily	Placebo	AF
PIONEER 6, 2019[45]	NCT02692716	T2DM with high CVD risk	3182	2176(68.4%)	66.0	32.3	8.2%	68 weeks	Oral semaglutide 14mg, daily	Placebo	AF/AFL/VAs/SCD
PIONEER 8, 2019[46]	NCT03021187	T2DM with insulin	730	395(54%)	61.0	31.0	8.2%	52 weeks	Oral semaglutide 3/7/14mg, daily	Placebo	AFL/VAs
SUSTAIN 6, 2016[47]	NCT01720446	T2DM with a standard care regimen	3297	2002(60.7%)	64.6	32.8	8.7%	104 weeks	Sc semaglutide 0.5mg/1mg, weekly	Placebo	AF/AFL/VAs/SCD
SUSTAIN 2, 2017[48]	NCT01930188	T2DM with OAD	1225	620(50.6%)	55.1	32.5	8.1%	56 weeks	Sc semaglutide 0.5mg/1mg, weekly	Sitagliptin, placebo	AF
SUSTAIN 4, 2017[49]	NCT02128932	T2DM with naïve insulin	1082	574(53%)	56.5	33.0	8.2%	30 weeks	Sc semaglutide 0.5mg/1mg, weekly	Insulin glargine	AF
Kaku et al, 2018[50]	NCT02207374	T2DM with OAD	600	429(71.5%)	58.5	26.4	8.1%	56 weeks	Sc semaglutide 0.5mg/1mg, weekly	Placebo	AF
SUSTAIN China, 2021[51]	NCT03061214	T2DM with metformin	867	499(57.5%)	53.1	28.0	8.1%	30 weeks	Sc semaglutide 0.5mg/1mg, weekly	Sitagliptin	AF/AFL
STEP 1, 2021[52]	NCT03548935	Overweight or obese, without T2DM	1961	508(24.9%)	46.0	37.8	5.7%	68 weeks	Sc semaglutide 2.4 mg, weekly	placebo	AF
STEP 2, 2021[53]	NCT03552757	Overweight or obesity	1207	594(49.1%)	55.0	35.7	8.1%	68 weeks	Sc semaglutide 1mg/ 2.4mg, weekly	placebo	AF
STEP 4, 2021[54]	NCT03548987	Overweight or obesity	803	169(21%)	46.0	34.1	5.4%	68 weeks	Sc semaglutide 2.4 mg, weekly	placebo	AF
STEP 6, 2022[55]	NCT03811574	Obesity, with or without T2DM	400	253(63.1%)	51.0	31.9	6.4%	68 weeks	Sc semaglutide 1.7mg/2.4 mg, weekly	placebo	AF

Trials, year	Registration number	Inclusion criteria	Population size	Males, n(%)	Age (years)	BMI (kg/m^2)	HbA1c	Follow-up duration	Interventions	Controls	Outcomes of interest
SUSTAIN 11, 2022[56]	NCT03689374	T2DM with insulin glargine and metformin	1728	894(51.1%)	61.2	31.5	8.6%	52 weeks	Sc semaglutide 1mg, weekly	Insulin Aspart	AF/AFL/SCD

Notes: The registration number represents the unique identifier of ClinicalTrials.gov or EudraCT. Abbreviations: GLP-1 RAs, glucagon-like peptide 1 receptor agonists; T2DM, Type 2 diabetes mellitus; OAD, oral anti-diabetic drugs; CVD, cardiovascular disease; CKD, chronic kidney disease; SGLT-2i, sodium-glucose cotransporter-2 inhibitors; BMI, body mass index; Sc, subcutaneous injection; NA, not available; AF, atrial fibrillation; AFL, atrial flutter; VAs, ventricular arrhythmias; SCD, sudden cardiac death.

Figure S1. Methodological quality assessment of included studies

Notes: Domain 1: Risk of bias arising from randomization process Domain 2: Risk of bias due to deviations from intended interventions Domain 3: Risk of bias due to missing outcome data. Domain 4: Risk of bias in outcome measurements. Domain 5: Risk of bias in the selection of reported results.

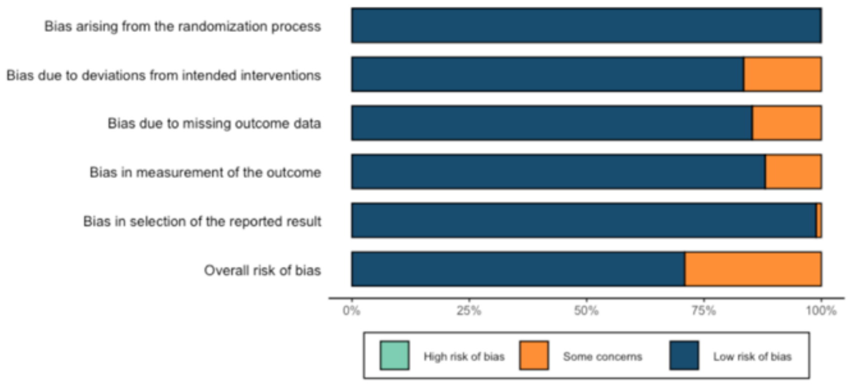

Figure S1.1. Summary of risk of bias of all included studies

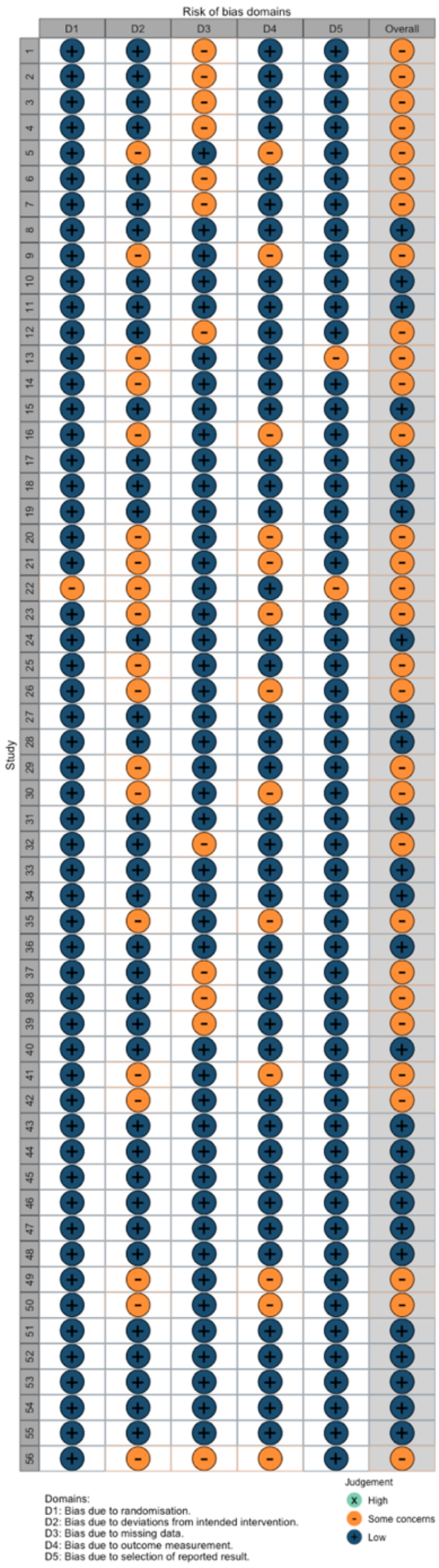

Figure S1.2. Risk of bias for each study

Figure S2. Risks of cardiac arrhythmias in patients with GLP-1 RAs treatments compared with control groups

Notes: Experimental, GLP-1-RAs treatments; Control, placebo or active control. Abbreviations: GLP-1 RAs, glucagon-like peptide 1 receptor agonists; RR, relative risk; CI, confidence interval.

Figure S2.1. The risk of atrial fibrillation in patients with GLP-1RAs

Study	GLP-1 RAs Events	GLP-1 RAs Total	Control Events	Control Total	RR	95%-CI	Weight
HARMONY 1, 2014	1	150	0	151	3.02	[0.12; 73.54]	0.1%
HARMONY 4, 2014	3	504	0	241	3.35	[0.17; 64.61]	0.2%
HARMONY 8, 2014	4	249	1	246	3.95	[0.44; 35.11]	0.3%
Rosenstock et al, 2014	2	285	0	281	4.93	[0.24; 102.23]	0.1%
HARMONY 5, 2015	1	271	1	392	1.45	[0.09; 23.03]	0.2%
HARMONY 2, 2016	1	200	0	101	1.52	[0.06; 36.95]	0.2%
Harmony Outcomes, 2018	45	4717	41	4715	1.10	[0.72; 1.67]	12.0%
Rosenstock et al, 2020	0	400	1	413	0.34	[0.01; 8.42]	0.4%
AWARD-3, 2014	0	539	1	268	0.17	[0.01; 4.06]	0.6%
AWARD-5, 2014	1	606	0	492	2.44	[0.10; 59.67]	0.2%
AWARD-4, 2015	1	588	0	296	1.51	[0.06; 36.99]	0.2%
AWARD-9, 2017	1	150	0	150	3.00	[0.12; 73.06]	0.1%
AWARD-7, 2018	1	382	0	194	1.53	[0.06; 37.27]	0.2%
AWARD-10, 2018	0	283	1	140	0.17	[0.01; 4.03]	0.6%
REWIND, 2019	90	4943	62	4949	1.45	[1.05; 2.00]	18.1%
Heine et al, 2005	1	282	0	267	2.84	[0.12; 69.43]	0.1%
Nauck et al, 2007	1	253	0	248	2.94	[0.12; 71.85]	0.1%
NCT00701935, 2008	0	43	1	37	0.29	[0.01; 6.85]	0.5%
EUREXA, 2012	2	511	2	508	0.99	[0.14; 7.03]	0.6%
Inagaki et al, 2012	1	215	0	212	2.96	[0.12; 72.21]	0.1%
LEAD-2, 2009	2	724	1	363	1.00	[0.09; 11.02]	0.4%
LEAD-3 Mono, 2009	0	498	1	248	0.17	[0.01; 4.06]	0.6%
Charbonnel et al, 2012	0	324	1	326	0.34	[0.01; 8.20]	0.4%
MDI-Liraglutide, 2015	0	64	1	60	0.31	[0.01; 7.53]	0.5%
SCALE Obesity and Prediabetes, 2015	6	2481	2	1242	1.50	[0.30; 7.43]	0.8%
Vanderheiden et al, 2016	1	35	0	36	3.08	[0.13; 73.22]	0.1%
LEADER, 2016	89	4668	97	4672	0.92	[0.69; 1.22]	28.3%
Zang et al, 2016	0	183	1	184	0.34	[0.01; 8.17]	0.4%
SCALE Insulin, 2020	0	195	1	197	0.34	[0.01; 8.22]	0.4%
GETGOAL-M, 2013	1	510	0	170	1.00	[0.04; 24.48]	0.2%
GETGOAL-L, 2013	0	328	1	167	0.17	[0.01; 4.15]	0.6%
GETGOAL-F1, 2014	2	322	1	160	0.99	[0.09; 10.88]	0.4%
ELIXA, 2015	23	3031	30	3032	0.77	[0.45; 1.32]	8.8%
GetGoal-Duo-2, 2016	0	298	1	595	0.66	[0.03; 16.27]	0.3%
PIONEER 2, 2019	0	410	3	409	0.14	[0.01; 2.75]	1.0%
PIONEER 3, 2019	6	1395	4	466	0.50	[0.14; 1.77]	1.8%
PIONEER 5, 2019	1	163	1	161	0.99	[0.06; 15.66]	0.3%
PIONEER 6, 2019	6	1591	14	1591	0.43	[0.17; 1.11]	4.1%
SUSTAIN 6, 2016	26	1648	38	1649	0.68	[0.42; 1.12]	11.1%
SUSTAIN 2, 2017	1	818	0	407	1.49	[0.06; 36.58]	0.2%
SUSTAIN 4, 2017	2	722	1	360	1.00	[0.09; 10.96]	0.4%
Kaku et al, 2018	1	480	0	120	0.75	[0.03; 18.35]	0.2%
SUSTAIN China, 2021	1	577	0	290	1.51	[0.06; 36.93]	0.2%
STEP 1, 2021	2	1306	1	655	1.00	[0.09; 11.04]	0.4%
STEP 2, 2021	4	805	2	402	1.00	[0.18; 5.43]	0.8%
STEP 4, 2021	1	535	2	268	0.25	[0.02; 2.75]	0.8%
STEP 6, 2022	0	299	1	101	0.11	[0.00; 2.75]	0.7%
SUSTAIN 11, 2022	1	874	3	864	0.33	[0.03; 3.16]	0.9%
**Common effect model**		**40855**		**33496**	**0.97**	**[0.83; 1.12]**	**100.0%**

Heterogeneity: $I^2 = 0\%$, $\tau^2 = 0.0428$, $p = 0.93$

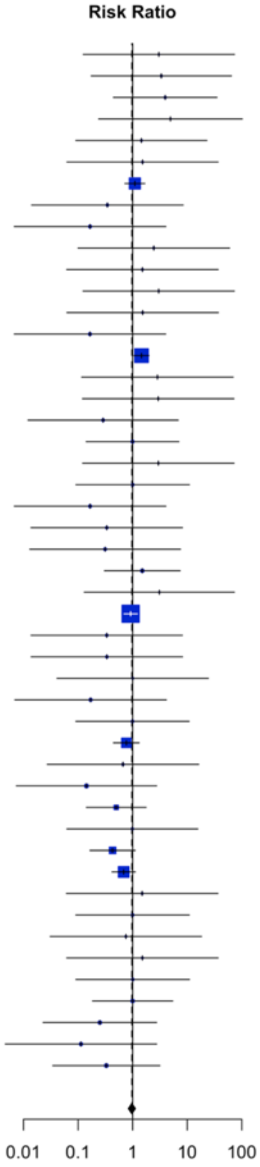

Figure S2.2. The risk of atrial flutter in patients with GLP-1RAs

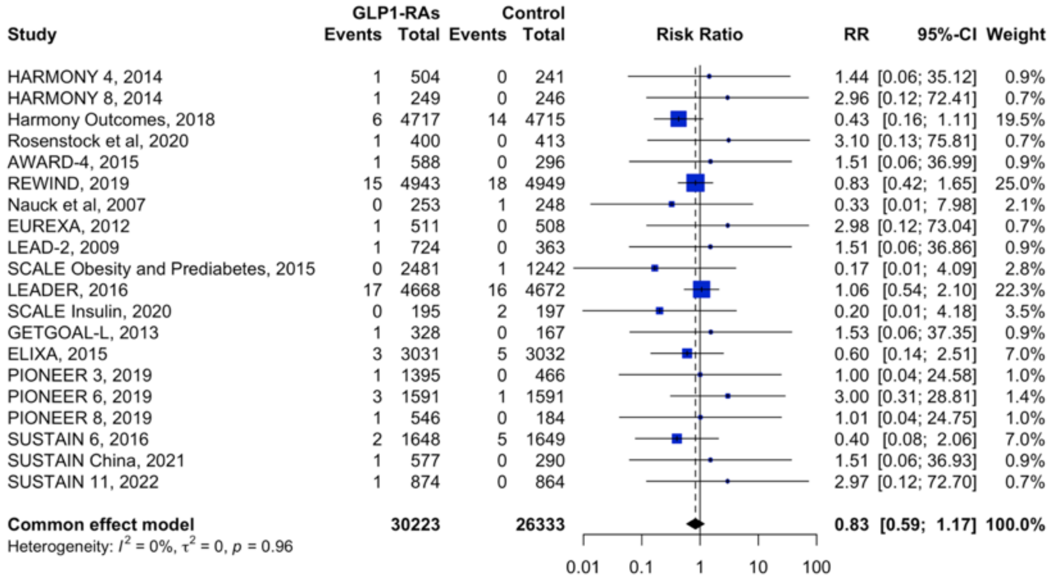

Figure S2.3. The risk of ventricular arrhythmias in patients with GLP-1RAs

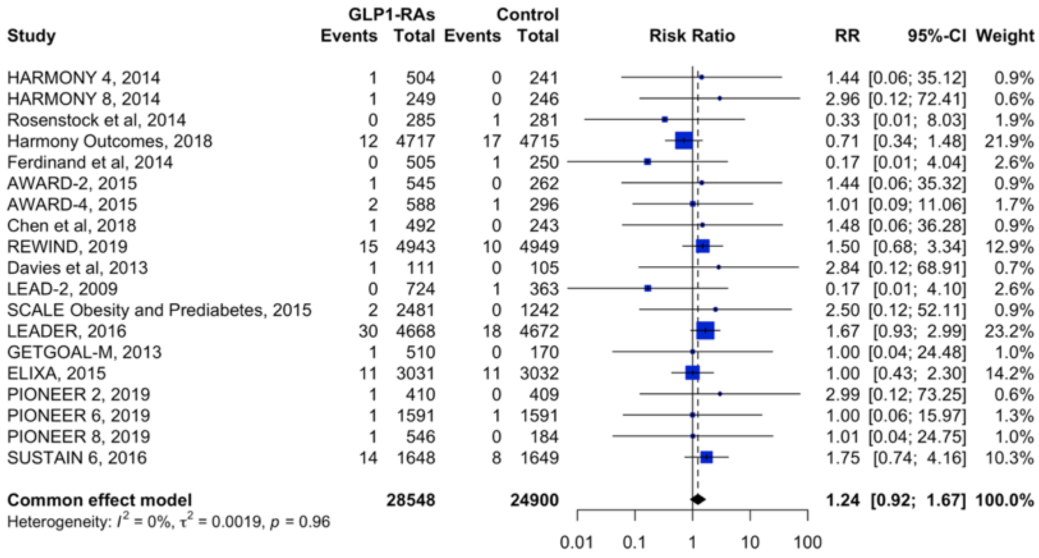

Figure S2.4. The risk of sudden cardiac death in patients with GLP-1RAs

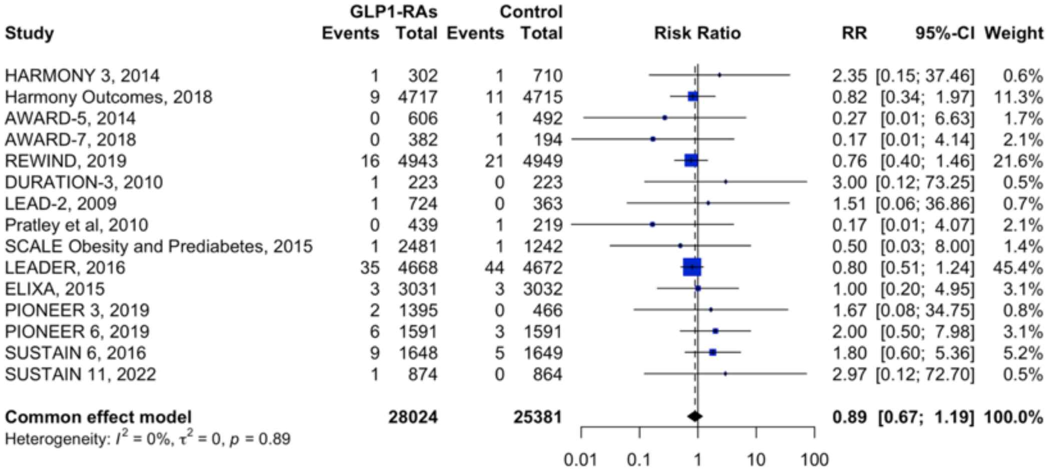

Figure S3. Subgroup analyses on the association of GLP-1 RAs use with incident atrial fibrillation

Notes: Experimental, GLP-1RAs treatments; Control, placebo or active control. Study designs: CVOT or non-CVOT. Abbreviations: RR, relative risks; CI, confidential intervals; GLP-1RAs, glucagon-like peptide 1 receptor agonists; BMI, body mass index; CVOT, cardiovascular outcome trial.

Figure S3.1. Effects of different GLP-1 RAs individuals on the association between GLP-1 RAs and the risk of atrial fibrillation

Study	GLP-1 RAs Events	GLP-1 RAs Total	Control Events	Control Total	RR	95%-CI	Weight
**Drug types = Albiglutide**							
HARMONY 1, 2014	1	150	0	151	3.02	[0.12; 73.54]	0.1%
HARMONY 4, 2014	3	504	0	241	3.35	[0.17; 64.61]	0.2%
HARMONY 8, 2014	4	249	1	246	3.95	[0.44; 35.11]	0.3%
Rosenstock et al, 2014	2	285	0	281	4.93	[0.24; 102.23]	0.1%
HARMONY 5, 2015	1	271	1	392	1.45	[0.09; 23.03]	0.2%
HARMONY 2, 2016	1	200	0	101	1.52	[0.06; 36.95]	0.2%
Harmony Outcomes, 2018	45	4717	41	4715	1.10	[0.72; 1.67]	12.0%
Rosenstock et al, 2020	0	400	1	413	0.34	[0.01; 8.42]	0.4%
Common effect model		6776		6540	1.24	[0.84; 1.83]	13.6%
Heterogeneity: $I^2 = 0\%$, $\tau^2 = 0$, $p = 0.83$							
Test for effect in subgroup: $z = 1.10$ ($p = 0.27$)							
**Drug types = Dulaglutide**							
AWARD-3, 2014	0	539	1	268	0.17	[0.01; 4.06]	0.6%
AWARD-5, 2014	1	606	0	492	2.44	[0.10; 59.67]	0.2%
AWARD-4, 2015	1	588	0	296	1.51	[0.06; 36.99]	0.2%
AWARD-9, 2017	1	150	0	150	3.00	[0.12; 73.06]	0.1%
AWARD-7, 2018	1	382	0	194	1.53	[0.06; 37.27]	0.2%
AWARD-10, 2018	0	283	1	140	0.17	[0.01; 4.03]	0.6%
REWIND, 2019	90	4943	62	4949	1.45	[1.05; 2.00]	18.1%
Common effect model		7491		6489	1.40	[1.03; 1.90]	20.0%
Heterogeneity: $I^2 = 0\%$, $\tau^2 = 0$, $p = 0.70$							
Test for effect in subgroup: $z = 2.14$ ($p = 0.03$)							
**Drug types = Exenatide**							
Heine et al, 2005	1	282	0	267	2.84	[0.12; 69.43]	0.1%
Nauck et al, 2007	1	253	0	248	2.94	[0.12; 71.85]	0.1%
NCT00701935, 2008	0	43	1	37	0.29	[0.01; 6.85]	0.5%
EUREXA, 2012	2	511	2	508	0.99	[0.14; 7.03]	0.6%
Inagaki et al, 2012	1	215	0	212	2.96	[0.12; 72.21]	0.1%
Common effect model		1304		1272	1.34	[0.43; 4.18]	1.5%
Heterogeneity: $I^2 = 0\%$, $\tau^2 = 0$, $p = 0.80$							
Test for effect in subgroup: $z = 0.51$ ($p = 0.61$)							
**Drug types = Liraglutide**							
LEAD-2, 2009	2	724	1	363	1.00	[0.09; 11.02]	0.4%
LEAD-3 Mono, 2009	0	498	1	248	0.17	[0.01; 4.06]	0.6%
Charbonnel et al, 2012	0	324	1	326	0.34	[0.01; 8.20]	0.4%
MDI-Liraglutide, 2015	0	64	1	60	0.31	[0.01; 7.53]	0.5%
SCALE Obesity and Prediabetes, 2015	6	2481	2	1242	1.50	[0.30; 7.43]	0.8%
Vanderheiden et al, 2016	1	35	0	36	3.08	[0.13; 73.22]	0.1%
LEADER, 2016	89	4668	97	4672	0.92	[0.69; 1.22]	28.3%
Zang et al, 2016	0	183	1	184	0.34	[0.01; 8.17]	0.4%
SCALE Insulin, 2020	0	195	1	197	0.34	[0.01; 8.22]	0.4%
Common effect model		9172		7328	0.90	[0.68; 1.17]	32.0%
Heterogeneity: $I^2 = 0\%$, $\tau^2 = 0$, $p = 0.89$							
Test for effect in subgroup: $z = -0.79$ ($p = 0.43$)							
**Drug types = Lixisenatide**							
GETGOAL-M, 2013	1	510	0	170	1.00	[0.04; 24.48]	0.2%
GETGOAL-L, 2013	0	328	1	167	0.17	[0.01; 4.15]	0.6%
GETGOAL-F1, 2014	2	322	1	160	0.99	[0.09; 10.88]	0.4%
ELIXA, 2015	23	3031	30	3032	0.77	[0.45; 1.32]	8.8%
GetGoal-Duo-2, 2016	0	298	1	595	0.66	[0.03; 16.27]	0.3%
Common effect model		4489		4124	0.74	[0.45; 1.23]	10.2%
Heterogeneity: $I^2 = 0\%$, $\tau^2 = 0$, $p = 0.92$							
Test for effect in subgroup: $z = -1.15$ ($p = 0.25$)							
**Drug types = Oral semaglutide**							
PIONEER 2, 2019	0	410	3	409	0.14	[0.01; 2.75]	1.0%
PIONEER 3, 2019	6	1395	4	466	0.50	[0.14; 1.77]	1.8%
PIONEER 5, 2019	1	163	1	161	0.99	[0.06; 15.66]	0.3%
PIONEER 6, 2019	6	1591	14	1591	0.43	[0.17; 1.11]	4.1%
Common effect model		3559		2627	0.43	[0.21; 0.87]	7.2%
Heterogeneity: $I^2 = 0\%$, $\tau^2 = 0$, $p = 0.82$							
Test for effect in subgroup: $z = -2.36$ ($p = 0.02$)							
**Drug types = Sc semaglutide**							
SUSTAIN 6, 2016	26	1648	38	1649	0.68	[0.42; 1.12]	11.1%
SUSTAIN 2, 2017	1	818	0	407	1.49	[0.06; 36.58]	0.2%
SUSTAIN 4, 2017	2	722	1	360	1.00	[0.09; 10.96]	0.4%
Kaku et al, 2018	1	480	0	120	0.75	[0.03; 18.35]	0.2%
SUSTAIN China, 2021	1	577	0	290	1.51	[0.06; 36.93]	0.2%
STEP 1, 2021	2	1306	1	655	1.00	[0.09; 11.04]	0.4%
STEP 2, 2021	4	805	2	402	1.00	[0.18; 5.43]	0.8%
STEP 4, 2021	1	535	2	268	0.25	[0.02; 2.75]	0.8%
STEP 6, 2022	0	299	1	101	0.11	[0.00; 2.75]	0.7%
SUSTAIN 11, 2022	1	874	3	864	0.33	[0.03; 3.16]	0.9%
Common effect model		8064		5116	0.67	[0.44; 1.02]	15.6%
Heterogeneity: $I^2 = 0\%$, $\tau^2 = 0$, $p = 0.96$							
Test for effect in subgroup: $z = -1.87$ ($p = 0.06$)							
**Common effect model**		**40855**		**33496**	**0.97**	**[0.83; 1.12]**	**100.0%**

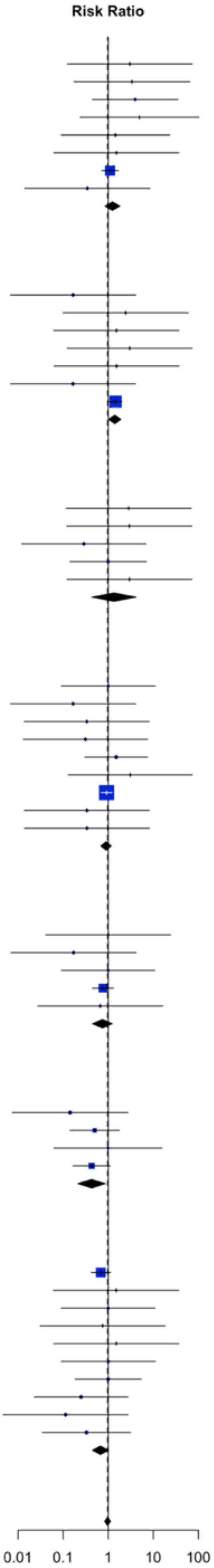

Heterogeneity: $I^2 = 0\%$, $\tau^2 = 0.0428$, $p = 0.93$
Test for overall effect: $z = -0.45$ ($p = 0.65$)
Test for subgroup differences: $\chi^2_6 = 16.86$, df = 6 ($p < 0.01$)

Figure S3.2. Effects of different treatment doses of GLP-1 RAs on the association between GLP-1 RAs and the risk of atrial fibrillation

Study	GLP-1 RAs Events	GLP-1 RAs Total	Control Events	Control Total	RR	95%-CI	Weight
Dose = Low dose							
HARMONY 1, 2014	1	150	0	151	3.02	[0.12; 73.54]	0.2%
HARMONY 4, 2014	3	504	0	241	3.35	[0.17; 64.61]	0.2%
HARMONY 8, 2014	4	249	1	246	3.95	[0.44; 35.11]	0.3%
Rosenstock et al, 2014	2	285	0	281	4.93	[0.24; 102.23]	0.2%
HARMONY 5, 2015	1	271	1	392	1.45	[0.09; 23.03]	0.3%
HARMONY 2, 2016	1	101	0	101	3.00	[0.12; 72.78]	0.2%
Harmony Outcomes, 2018	45	4717	41	4715	1.10	[0.72; 1.67]	12.8%
Rosenstock et al, 2020	0	400	1	413	0.34	[0.01; 8.42]	0.5%
AWARD-3, 2014	0	270	1	268	0.33	[0.01; 8.09]	0.5%
AWARD-5, 2014	1	302	0	492	4.88	[0.20; 119.51]	0.1%
AWARD-10, 2018	0	141	1	140	0.33	[0.01; 8.06]	0.5%
LEAD-2, 2009	0	482	1	363	0.25	[0.01; 6.15]	0.5%
LEAD-3 Mono, 2009	0	247	1	248	0.33	[0.01; 8.18]	0.5%
Charbonnel et al, 2012	0	324	1	326	0.34	[0.01; 8.20]	0.5%
PIONEER 3, 2019	3	466	4	466	0.75	[0.17; 3.33]	1.2%
SUSTAIN 6, 2016	14	826	38	1749	0.78	[0.43; 1.43]	7.6%
SUSTAIN 2, 2017	1	409	0	407	2.99	[0.12; 73.07]	0.2%
SUSTAIN 4, 2017	1	362	1	360	0.99	[0.06; 15.84]	0.3%
Kaku et al, 2018	1	239	0	120	1.51	[0.06; 36.78]	0.2%
Common effect model		10745		11479	1.04	[0.77; 1.40]	26.6%
Heterogeneity: I^2 = 0%, τ^2 = 0, p = 0.94							
Test for effect in subgroup: z = 0.23 (p = 0.81)							
Dose = High dose							
AWARD-3, 2014	0	269	1	268	0.33	[0.01; 8.12]	0.5%
AWARD-4, 2015	1	295	0	296	3.01	[0.12; 73.59]	0.2%
AWARD-9, 2017	1	150	0	150	3.00	[0.12; 73.06]	0.2%
AWARD-7, 2018	1	192	0	194	3.03	[0.12; 73.95]	0.2%
AWARD-10, 2018	0	142	1	140	0.33	[0.01; 8.00]	0.5%
REWIND, 2019	90	4943	62	4949	1.45	[1.05; 2.00]	19.4%
LEAD-2, 2009	2	242	1	363	3.00	[0.27; 32.90]	0.2%
LEAD-3 Mono, 2009	0	251	1	248	0.33	[0.01; 8.05]	0.5%
MDI-Liraglutide, 2015	0	64	1	60	0.31	[0.01; 7.53]	0.5%
SCALE Obesity and Prediabetes, 2015	6	2481	2	1242	1.50	[0.30; 7.43]	0.8%
Vanderheiden et al, 2016	1	35	0	36	3.08	[0.13; 73.22]	0.2%
LEADER, 2016	89	4668	97	4672	0.92	[0.69; 1.22]	30.3%
Zang et al, 2016	0	183	1	184	0.34	[0.01; 8.17]	0.5%
SCALE Insulin, 2020	0	195	1	197	0.34	[0.01; 8.22]	0.5%
PIONEER 2, 2019	0	410	3	409	0.14	[0.01; 2.75]	1.1%
PIONEER 3, 2019	3	929	4	466	0.38	[0.08; 1.67]	1.7%
PIONEER 5, 2019	1	163	1	161	0.99	[0.06; 15.66]	0.3%
PIONEER 6, 2019	6	1591	14	1591	0.43	[0.17; 1.11]	4.4%
SUSTAIN 6, 2016	12	822	38	1749	0.67	[0.35; 1.28]	7.6%
SUSTAIN 4, 2017	1	360	1	360	1.00	[0.06; 15.93]	0.3%
SUSTAIN China, 2021	1	290	0	290	3.00	[0.12; 73.34]	0.2%
STEP 1, 2021	2	1306	1	655	1.00	[0.09; 11.04]	0.4%
STEP 2, 2021	4	805	2	402	1.00	[0.18; 5.43]	0.8%
STEP 4, 2021	1	535	2	268	0.25	[0.02; 2.75]	0.8%
STEP 6, 2022	0	299	1	101	0.11	[0.00; 2.75]	0.7%
SUSTAIN 11, 2022	1	874	3	864	0.33	[0.03; 3.16]	0.9%
Common effect model		22494		20315	0.97	[0.81; 1.17]	73.4%
Heterogeneity: I^2 = 0%, τ^2 = 0.0951, p = 0.55							
Test for effect in subgroup: z = -0.29 (p = 0.77)							
**Common effect model**		**33239**		**31794**	**0.99**	**[0.85; 1.16]**	**100.0%**
Heterogeneity: I^2 = 0%, τ^2 = 0.0400, p = 0.88							
Test for overall effect: z = -0.13 (p = 0.90)							
Test for subgroup differences: χ^2_1 = 0.12, df = 1 (p = 0.72)							

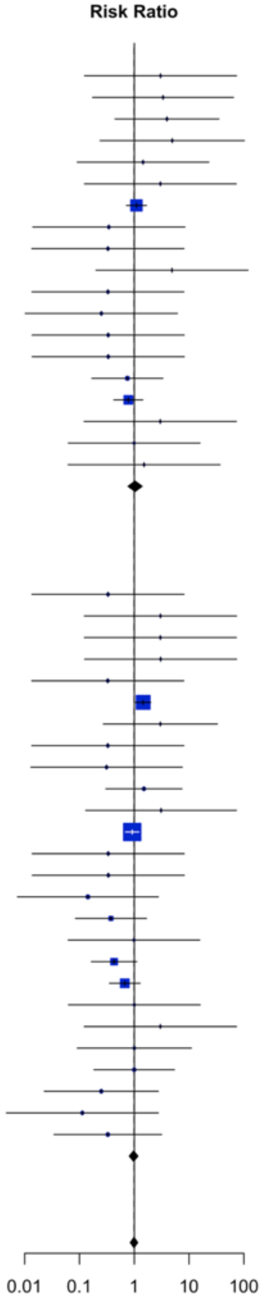

Figure S3.3. Effects of follow-up duration on the association between GLP-1 RAs and the risk of atrial fibrillation

Study	GLP-1 RAs Events	GLP-1 RAs Total	Control Events	Control Total	RR	95%-CI	Weight
**Follow-up duration = ≤52 weeks**							
HARMONY 1, 2014	1	150	0	151	3.02	[0.12; 73.54]	0.1%
HARMONY 4, 2014	3	504	0	241	3.35	[0.17; 64.61]	0.2%
HARMONY 8, 2014	4	249	1	246	3.95	[0.44; 35.11]	0.3%
Rosenstock et al, 2014	2	285	0	281	4.93	[0.24; 102.23]	0.1%
HARMONY 5, 2015	1	271	1	392	1.45	[0.09; 23.03]	0.2%
HARMONY 2, 2016	1	200	0	101	1.52	[0.06; 36.95]	0.2%
Rosenstock et al, 2020	0	400	1	413	0.34	[0.01; 8.42]	0.4%
AWARD-3, 2014	0	539	1	268	0.17	[0.01; 4.06]	0.6%
AWARD-5, 2014	1	606	0	492	2.44	[0.10; 59.67]	0.2%
AWARD-4, 2015	1	588	0	296	1.51	[0.06; 36.99]	0.2%
AWARD-9, 2017	1	150	0	150	3.00	[0.12; 73.06]	0.1%
AWARD-7, 2018	1	382	0	194	1.53	[0.06; 37.27]	0.2%
AWARD-10, 2018	0	283	1	140	0.17	[0.01; 4.03]	0.6%
Heine et al, 2005	1	282	0	267	2.84	[0.12; 69.43]	0.1%
Nauck et al, 2007	1	253	0	248	2.94	[0.12; 71.85]	0.1%
NCT00701935, 2008	0	43	1	37	0.29	[0.01; 6.85]	0.5%
Inagaki et al, 2012	1	215	0	212	2.96	[0.12; 72.21]	0.1%
LEAD-2, 2009	2	724	1	363	1.00	[0.09; 11.02]	0.4%
Charbonnel et al, 2012	0	324	1	326	0.34	[0.01; 8.20]	0.4%
MDI-Liraglutide, 2015	0	64	1	60	0.31	[0.01; 7.53]	0.5%
Vanderheiden et al, 2016	1	35	0	36	3.08	[0.13; 73.22]	0.1%
Zang et al, 2016	0	183	1	184	0.34	[0.01; 8.17]	0.4%
GETGOAL-M, 2013	1	510	0	170	1.00	[0.04; 24.48]	0.2%
GETGOAL-L, 2013	0	328	1	167	0.17	[0.01; 4.15]	0.6%
GETGOAL-F1, 2014	2	322	1	160	0.99	[0.09; 10.88]	0.4%
ELIXA, 2015	23	3031	30	3032	0.77	[0.45; 1.32]	8.8%
GetGoal-Duo-2, 2016	0	298	1	595	0.66	[0.03; 16.27]	0.3%
PIONEER 2, 2019	0	410	3	409	0.14	[0.01; 2.75]	1.0%
PIONEER 5, 2019	1	163	1	161	0.99	[0.06; 15.66]	0.3%
SUSTAIN 4, 2017	2	722	1	360	1.00	[0.09; 10.96]	0.4%
SUSTAIN China, 2021	1	577	0	290	1.51	[0.06; 36.93]	0.2%
SUSTAIN 11, 2022	1	874	3	864	0.33	[0.03; 3.16]	0.9%
Common effect model		13965		11306	0.89	[0.62; 1.26]	19.3%
Heterogeneity: $I^2 = 0\%$, $\tau^2 = 0$, $p = 0.99$							
Test for effect in subgroup: $z = -0.68$ ($p = 0.50$)							
**Follow-up duration = >52 weeks**							
Harmony Outcomes, 2018	45	4717	41	4715	1.10	[0.72; 1.67]	12.0%
REWIND, 2019	90	4943	62	4949	1.45	[1.05; 2.00]	18.1%
EUREXA, 2012	2	511	2	508	0.99	[0.14; 7.03]	0.6%
LEAD-3 Mono, 2009	0	498	1	248	0.17	[0.01; 4.06]	0.6%
SCALE Obesity and Prediabetes, 2015	6	2481	2	1242	1.50	[0.30; 7.43]	0.8%
LEADER, 2016	89	4668	97	4672	0.92	[0.69; 1.22]	28.3%
SCALE Insulin, 2020	0	195	1	197	0.34	[0.01; 8.22]	0.4%
PIONEER 3, 2019	6	1395	4	466	0.50	[0.14; 1.77]	1.8%
PIONEER 6, 2019	6	1591	14	1591	0.43	[0.17; 1.11]	4.1%
SUSTAIN 6, 2016	26	1648	38	1649	0.68	[0.42; 1.12]	11.1%
SUSTAIN 2, 2017	1	818	0	407	1.49	[0.06; 36.58]	0.2%
Kaku et al, 2018	1	480	0	120	0.75	[0.03; 18.35]	0.2%
STEP 1, 2021	2	1306	1	655	1.00	[0.09; 11.04]	0.4%
STEP 2, 2021	4	805	2	402	1.00	[0.18; 5.43]	0.8%
STEP 4, 2021	1	535	2	268	0.25	[0.02; 2.75]	0.8%
STEP 6, 2022	0	299	1	101	0.11	[0.00; 2.75]	0.7%
Common effect model		26890		22190	0.99	[0.84; 1.16]	80.7%
Heterogeneity: $I^2 = 13\%$, $\tau^2 = 0.0701$, $p = 0.30$							
Test for effect in subgroup: $z = -0.17$ ($p = 0.86$)							
**Common effect model**		**40855**		**33496**	**0.97**	**[0.83; 1.12]**	**100.0%**
Heterogeneity: $I^2 = 0\%$, $\tau^2 = 0.0428$, $p = 0.93$							
Test for overall effect: $z = -0.45$ ($p = 0.65$)							
Test for subgroup differences: $\chi^2_1 = 0.29$, df = 1 ($p = 0.59$)							

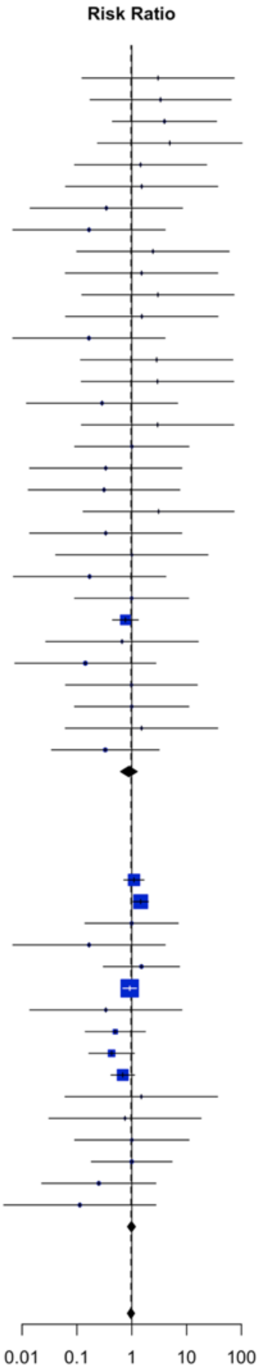

Figure S3.4. Effects of baseline BMI on the association between GLP-1 RAs and the risk of atrial fibrillation

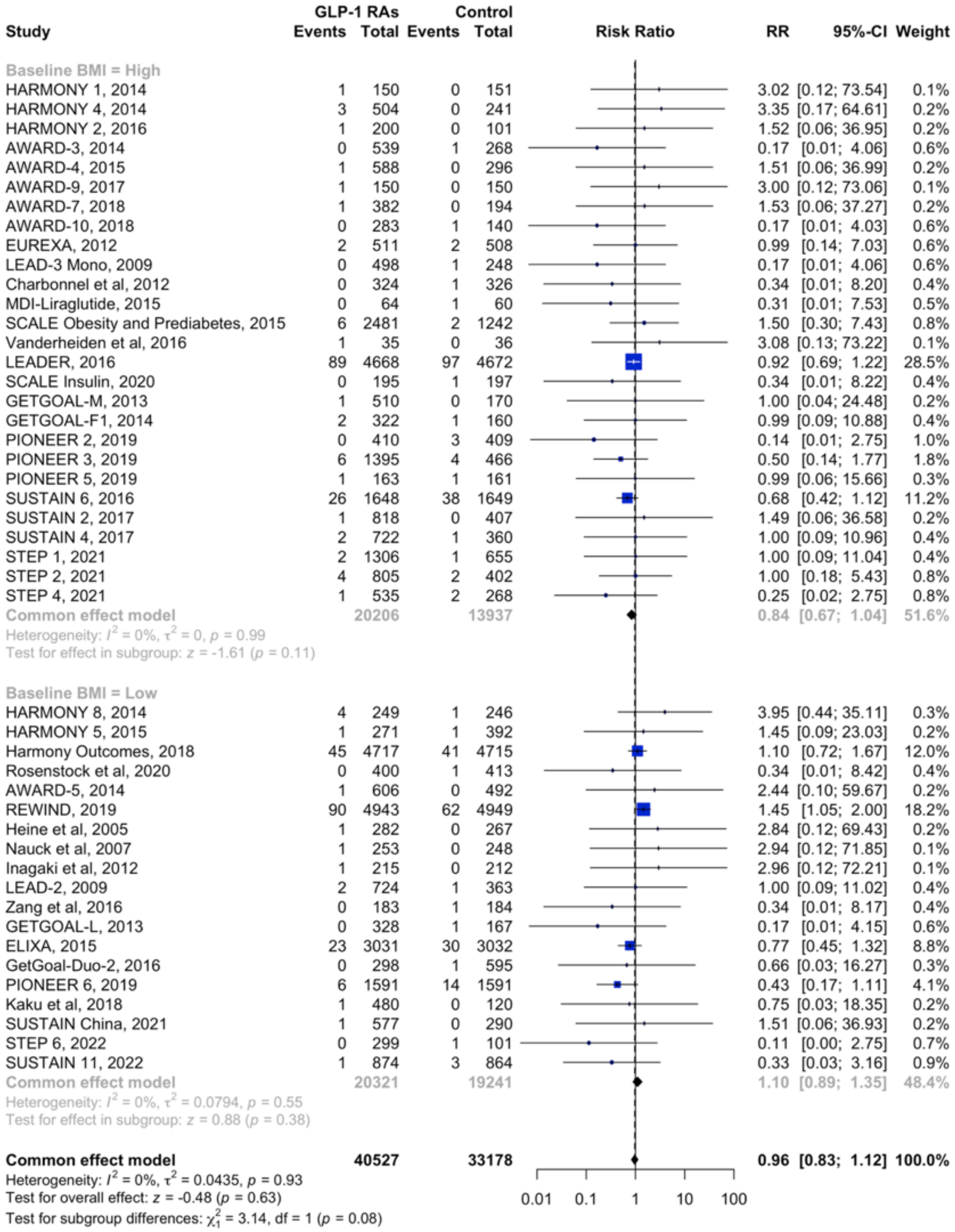

Figure S3.5. Effects of study designs on the association between GLP-1 RAs and the risk of atrial fibrillation

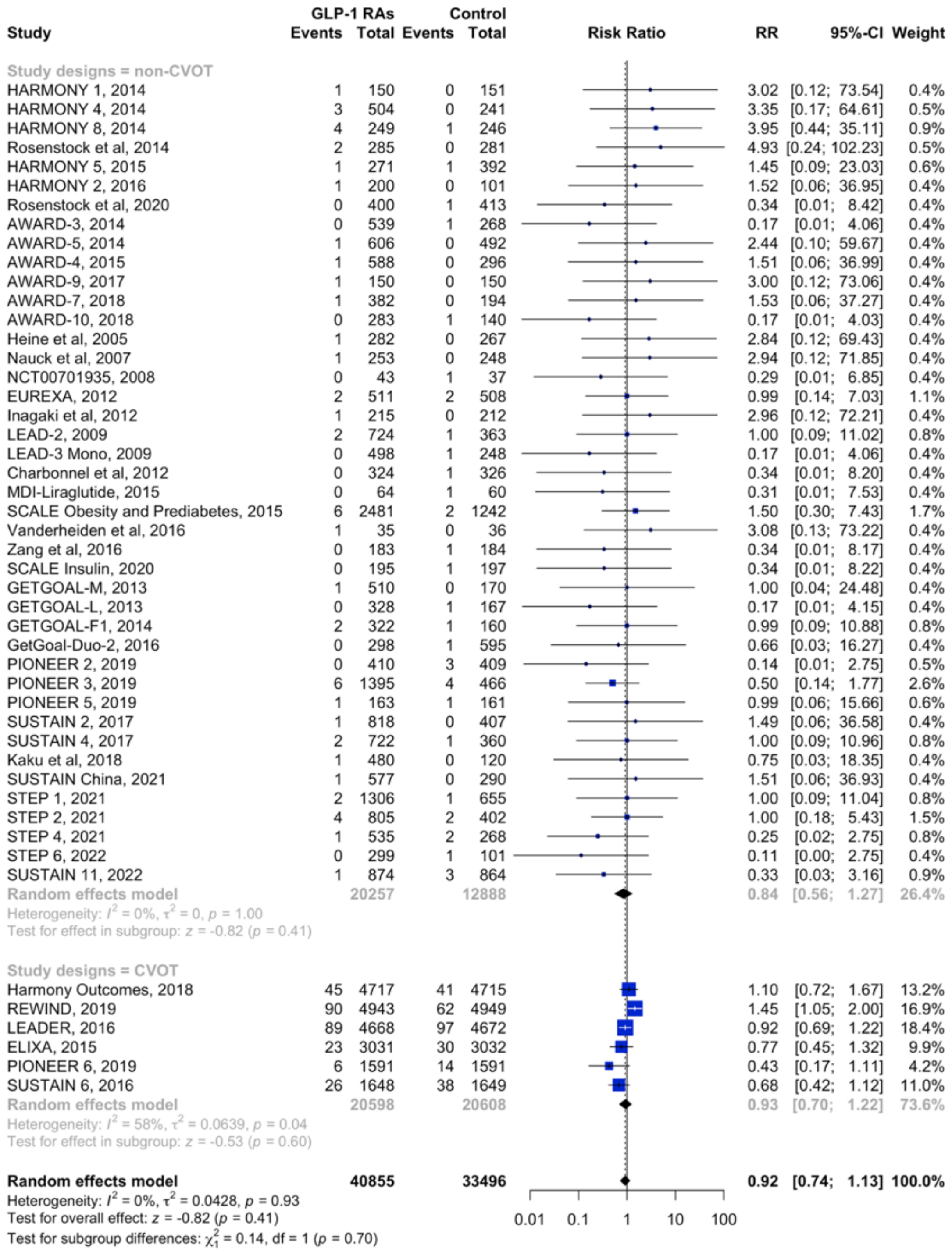

Figure S4. Subgroup analyses on the association of GLP-1 RAs use with the incidence of atrial flutter

Notes: Experimental, GLP-1RAs treatments; Control, placebo or active control. Study designs: CVOT or non-CVOT. Abbreviations: RR, relative risks; CI, confidential intervals; GLP-1RAs, glucagon-like peptide 1 receptor agonists; BMI, body mass index; CVOT, cardiovascular outcome trial.

Figure S4.1. Effects of different types of GLP-1 RAs medications on the association between GLP-1 RAs and the risk of atrial flutter

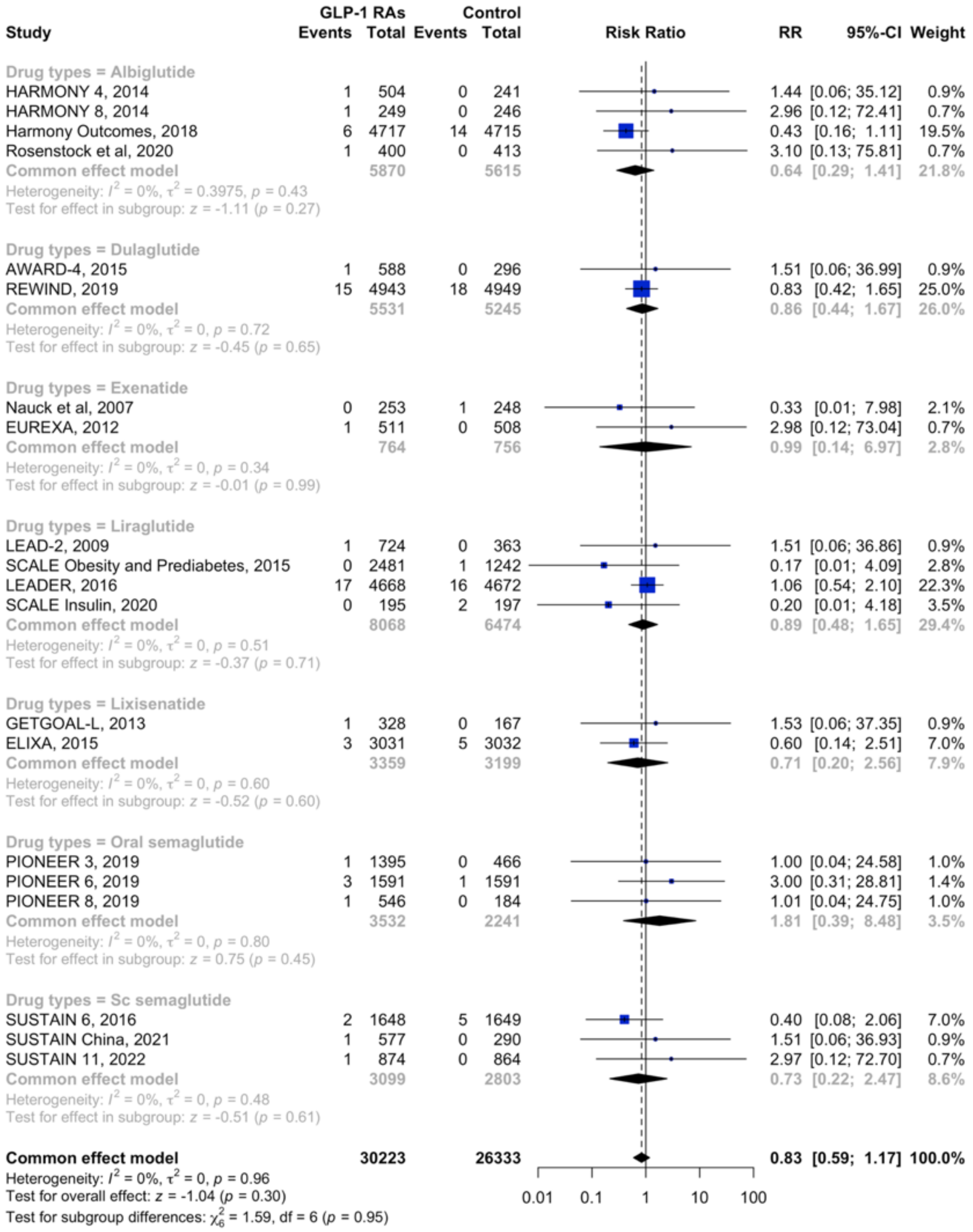

Figure S4.2. Effects of different doses of GLP-1 RAs medications on the association between GLP-1 RAs and the risk of atrial flutter

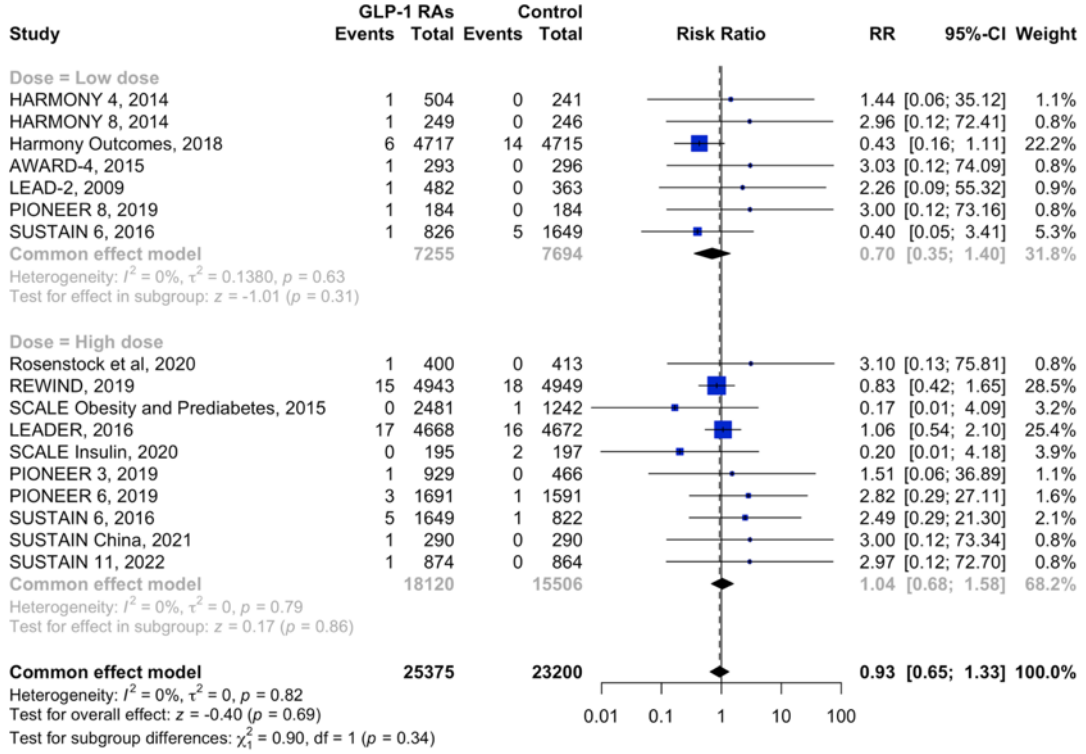

Figure S4.3. Effects of follow-up duration on the association between GLP-1 RAs and the risk of atrial flutter

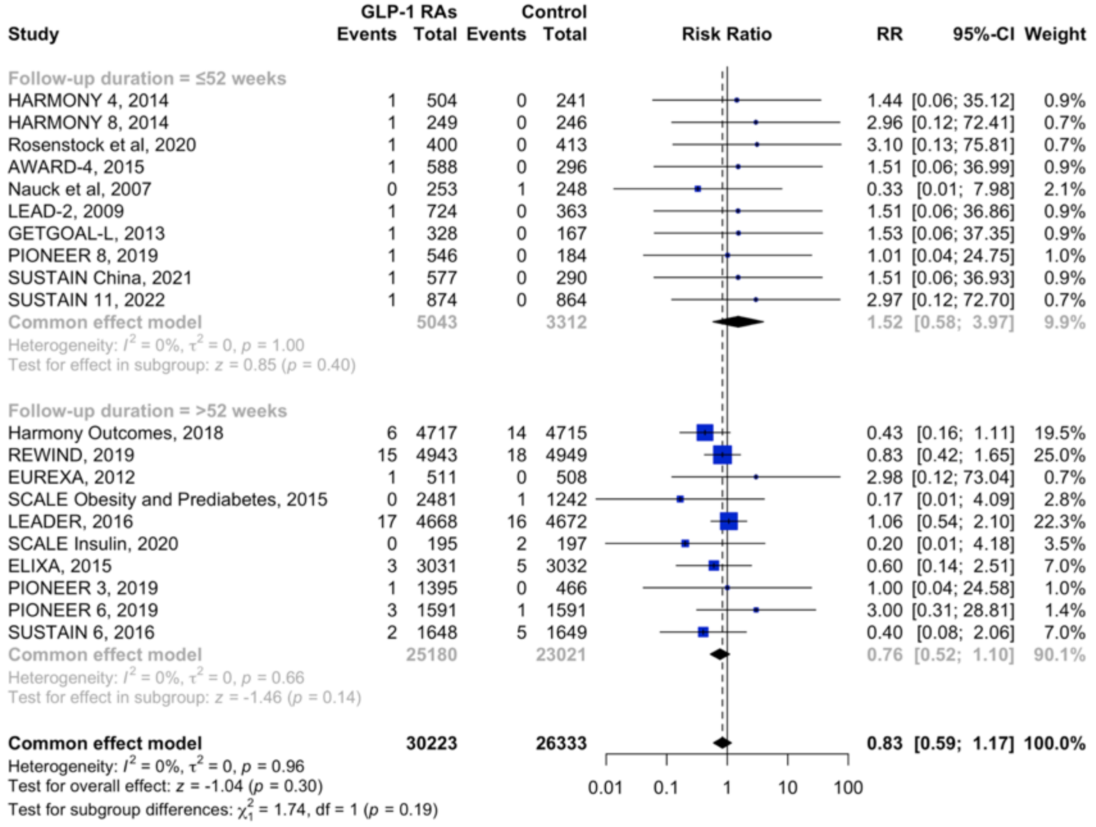

Figure S4.4. Effects of baseline BMI on the association between GLP-1 RAs and the risk of atrial flutter

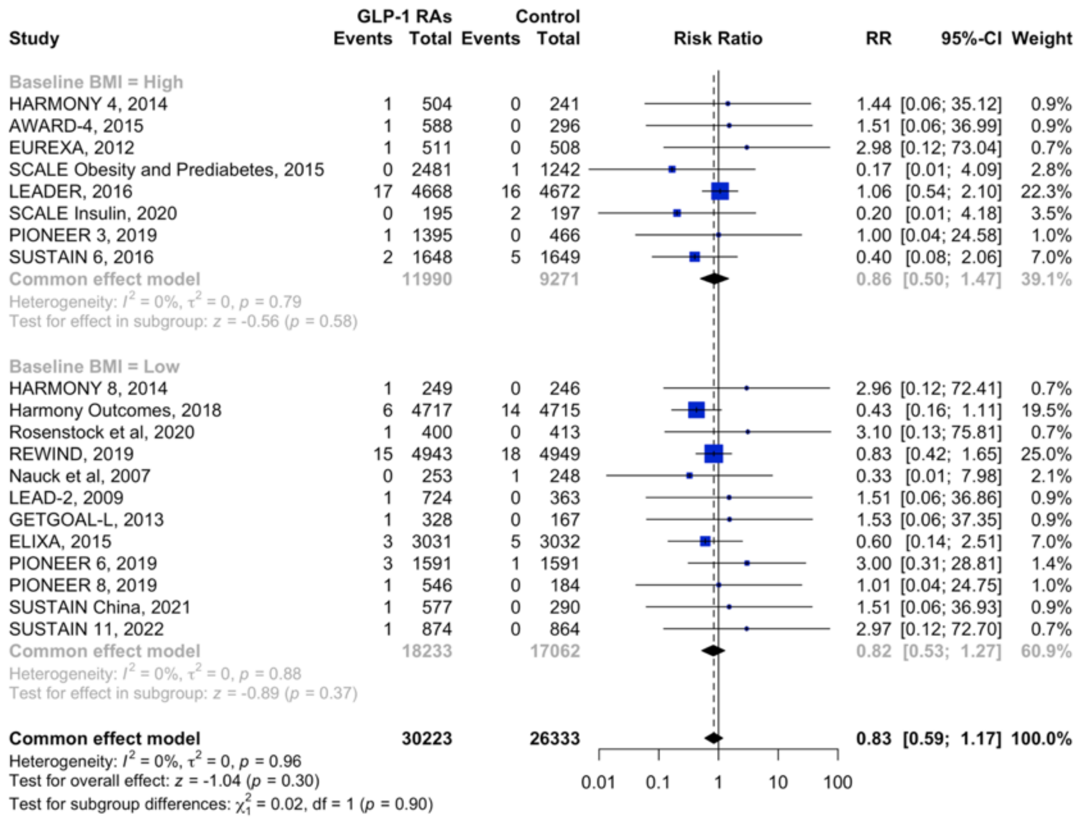

Figure S4.5. Effects of study designs on the association between GLP-1 RAs and the risk of atrial flutter

Study	GLP-1 RAs Events	GLP-1 RAs Total	Control Events	Control Total	RR	95%-CI	Weight
**Study designs = non-CVOT**							
HARMONY 4, 2014	1	504	0	241	1.44	[0.06; 35.12]	0.9%
HARMONY 8, 2014	1	249	0	246	2.96	[0.12; 72.41]	0.7%
Rosenstock et al, 2020	1	400	0	413	3.10	[0.13; 75.81]	0.7%
AWARD-4, 2015	1	588	0	296	1.51	[0.06; 36.99]	0.9%
Nauck et al, 2007	0	253	1	248	0.33	[0.01; 7.98]	2.1%
EUREXA, 2012	1	511	0	508	2.98	[0.12; 73.04]	0.7%
LEAD-2, 2009	1	724	0	363	1.51	[0.06; 36.86]	0.9%
SCALE Obesity and Prediabetes, 2015	0	2481	1	1242	0.17	[0.01; 4.09]	2.8%
SCALE Insulin, 2020	0	195	2	197	0.20	[0.01; 4.18]	3.5%
GETGOAL-L, 2013	1	328	0	167	1.53	[0.06; 37.35]	0.9%
PIONEER 3, 2019	1	1395	0	466	1.00	[0.04; 24.58]	1.0%
PIONEER 8, 2019	1	546	0	184	1.01	[0.04; 24.75]	1.0%
SUSTAIN China, 2021	1	577	0	290	1.51	[0.06; 36.93]	0.9%
SUSTAIN 11, 2022	1	874	0	864	2.97	[0.12; 72.70]	0.7%
Common effect model		9625		5725	1.08	[0.51; 2.30]	17.9%
Heterogeneity: $I^2 = 0\%$, $\tau^2 = 0$, $p = 0.98$							
Test for effect in subgroup: $z = 0.20$ ($p = 0.84$)							
**Study designs = CVOT**							
Harmony Outcomes, 2018	6	4717	14	4715	0.43	[0.16; 1.11]	19.5%
REWIND, 2019	15	4943	18	4949	0.83	[0.42; 1.65]	25.0%
LEADER, 2016	17	4668	16	4672	1.06	[0.54; 2.10]	22.3%
ELIXA, 2015	3	3031	5	3032	0.60	[0.14; 2.51]	7.0%
PIONEER 6, 2019	3	1591	1	1591	3.00	[0.31; 28.81]	1.4%
SUSTAIN 6, 2016	2	1648	5	1649	0.40	[0.08; 2.06]	7.0%
Common effect model		20598		20608	0.78	[0.53; 1.15]	82.1%
Heterogeneity: $I^2 = 0\%$, $\tau^2 = < 0.0001$, $p = 0.48$							
Test for effect in subgroup: $z = -1.26$ ($p = 0.21$)							
**Common effect model**		**30223**		**26333**	**0.83**	**[0.59; 1.17]**	**100.0%**
Heterogeneity: $I^2 = 0\%$, $\tau^2 = 0$, $p = 0.96$							
Test for overall effect: $z = -1.04$ ($p = 0.30$)							
Test for subgroup differences: $\chi^2_1 = 0.56$, df = 1 ($p = 0.46$)							

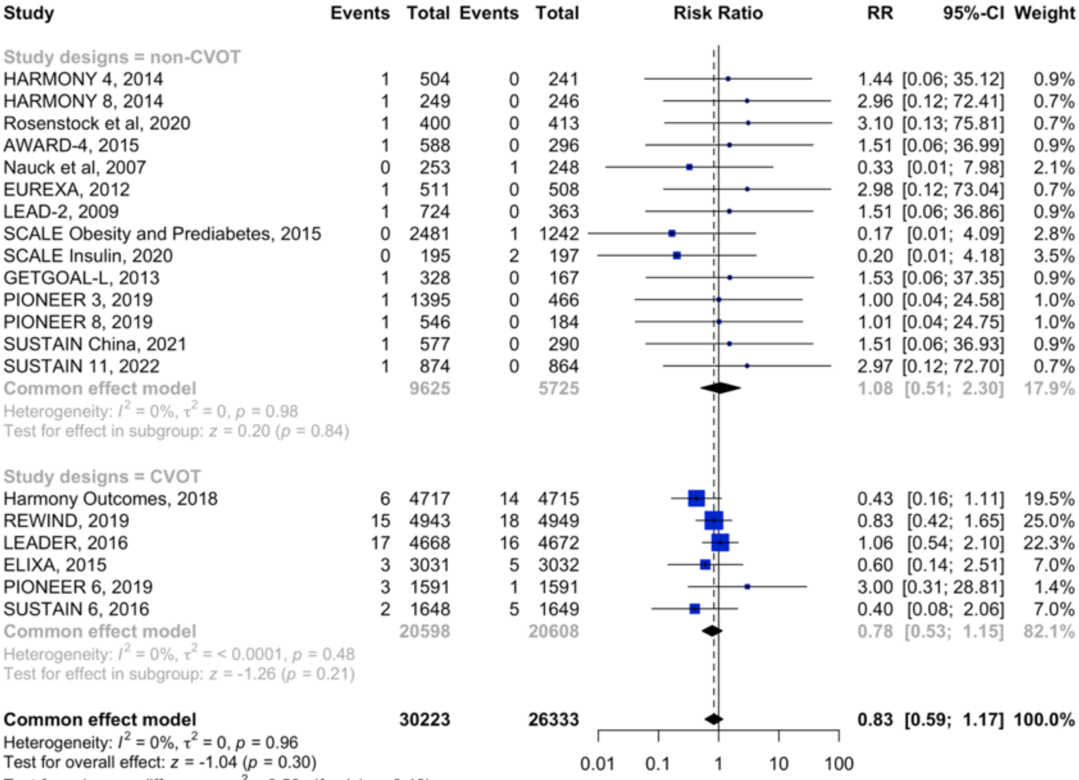

Figure S5. Subgroup analyses on the association of GLP-1 RAs use with the incidence of ventricular arrhythmias

Notes: Experimental, GLP-1RAs treatments; Control, placebo or active control. Study designs: CVOT or non-CVOT. Abbreviations: RR, relative risks; CI, confidential intervals; GLP-1RAs, glucagon-like peptide 1 receptor agonists; BMI, body mass index; CVOT, cardiovascular outcome trial.

Figure S5.1. Effects of different GLP-1 RAs individuals on the association between GLP-1 RAs and the risk of ventricular arrhythmias

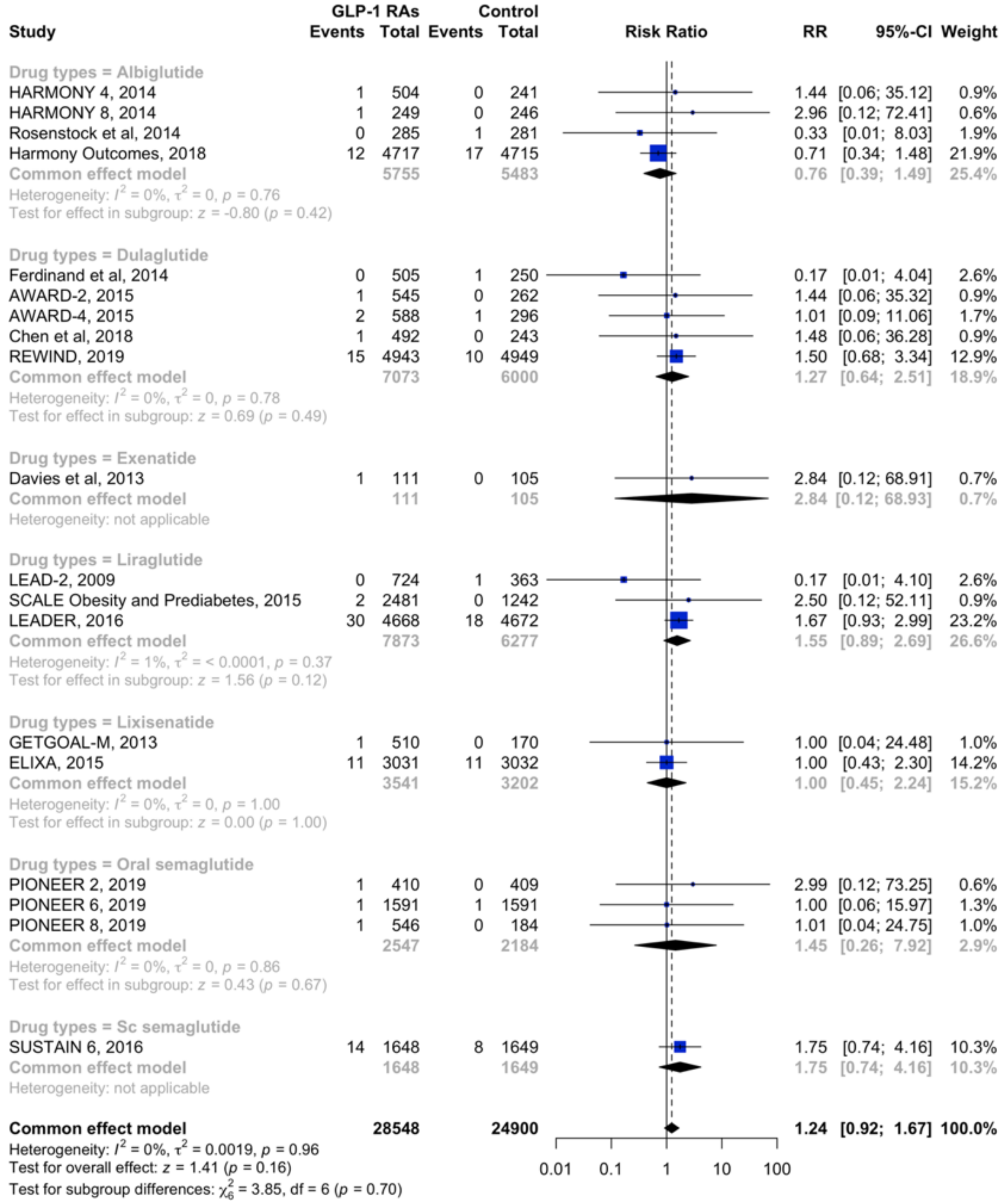

Figure S5.2. Effects of treatment doses of GLP-1 RAs medications on the association between GLP-1 RAs and the risk of ventricular arrhythmias

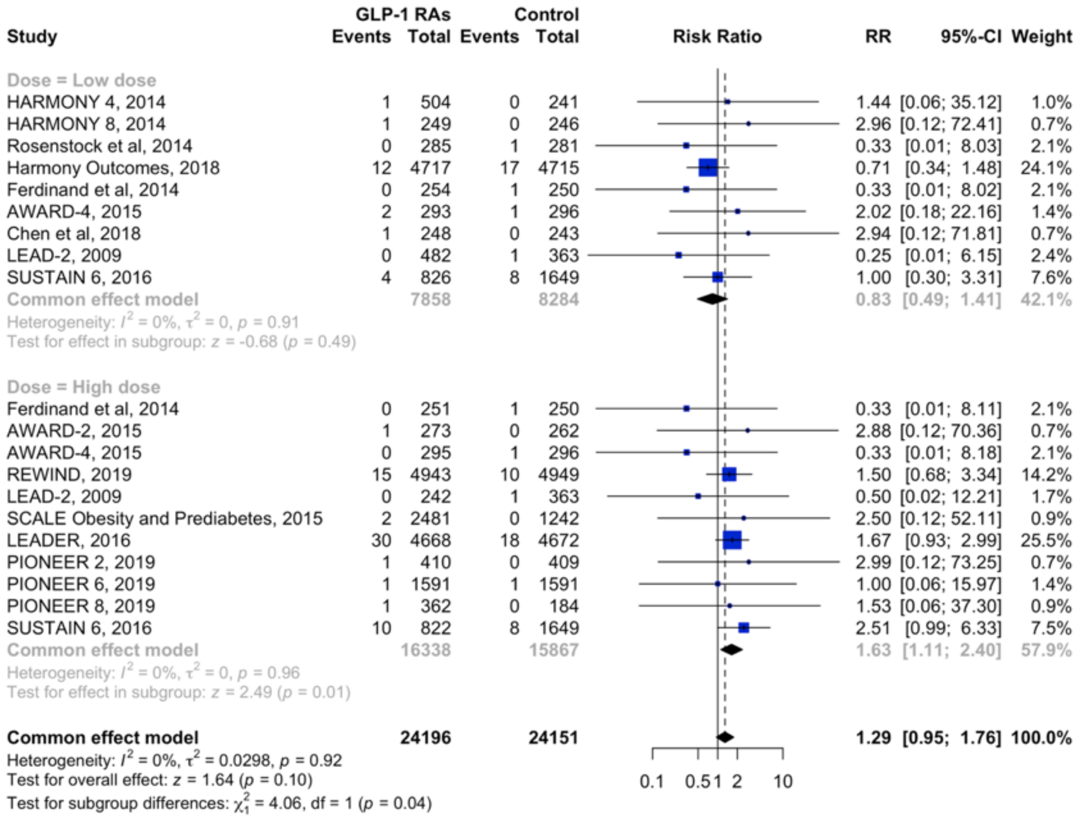

Figure S5.3. Effects of follow-up duration on the association between GLP-1 RAs and the risk of ventricular arrhythmias

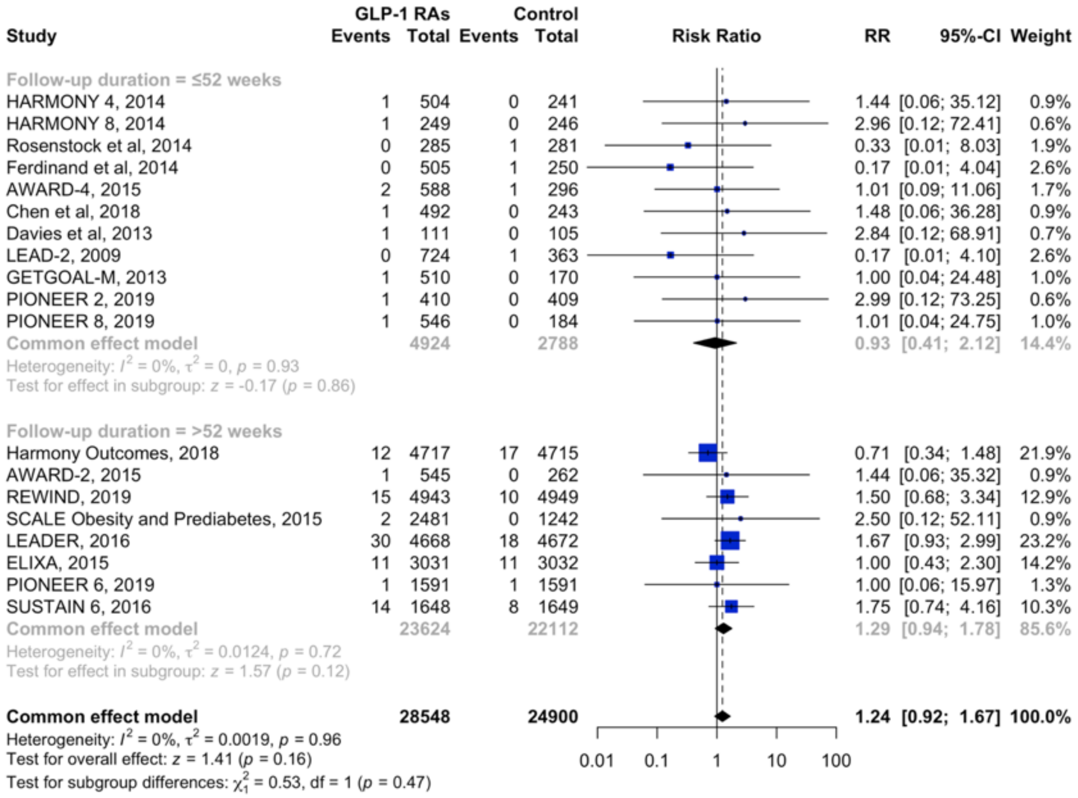

Figure S5.4. Effects of baseline BMI on the association between GLP-1 RAs and the risk of ventricular arrhythmias

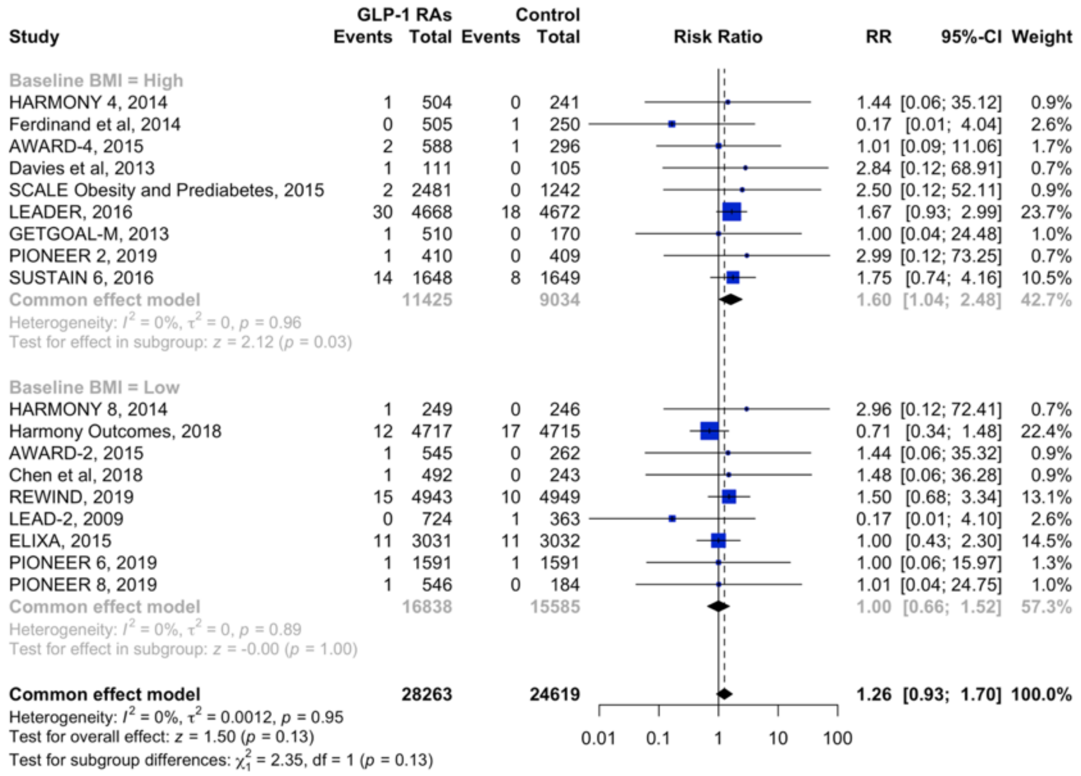

Figure S5.5. Effects of study designs on the association between GLP-1 RAs and the risk of ventricular arrhythmias

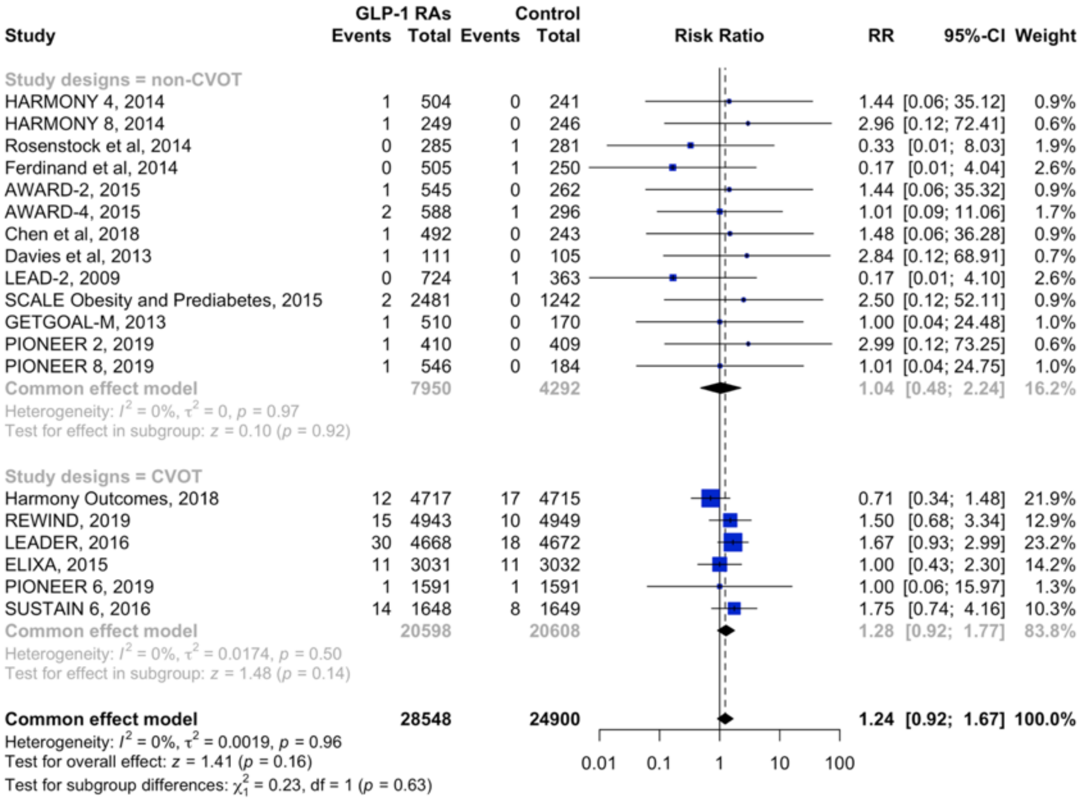

Figure S6. Subgroup analyses on the association of GLP-1 RAs use with the incidence of sudden cardiac death

Notes: Experimental, GLP-1RAs treatments; Control, placebo or active control. Study designs: CVOT or non-CVOT. Abbreviations: RR, relative risks; CI, confidential intervals; GLP-1RAs, glucagon-like peptide 1 receptor agonists; BMI, body mass index; CVOT, cardiovascular outcome trial.

Figure S6.1. Effects of different types of GLP-1 RAs medications on the association between GLP-1 RAs and the risk of sudden cardiac death

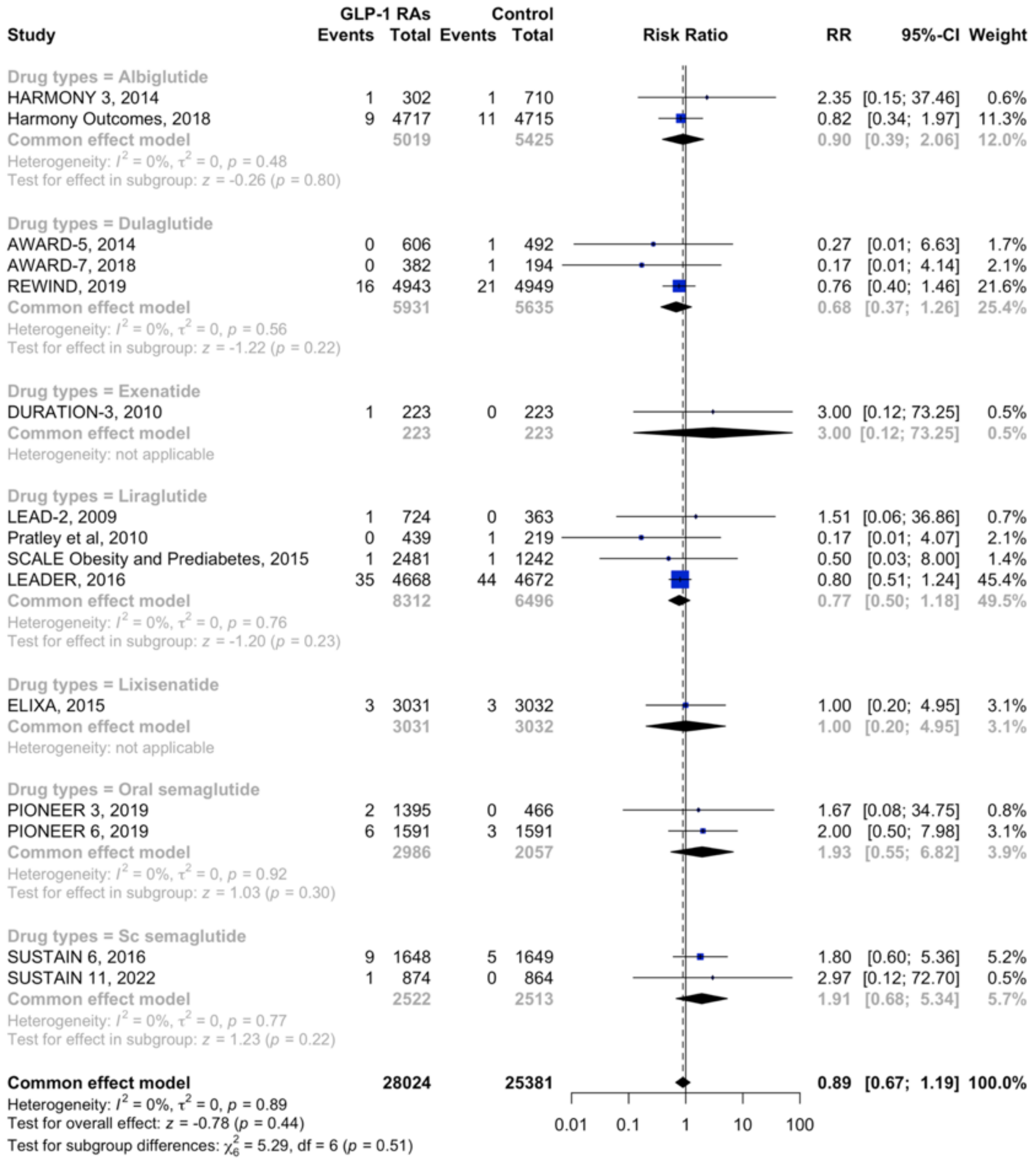

Figure S6.2. Effects of treatment doses of GLP-1 RAs medications on the association between GLP-1 RAs and the risk of sudden cardiac death

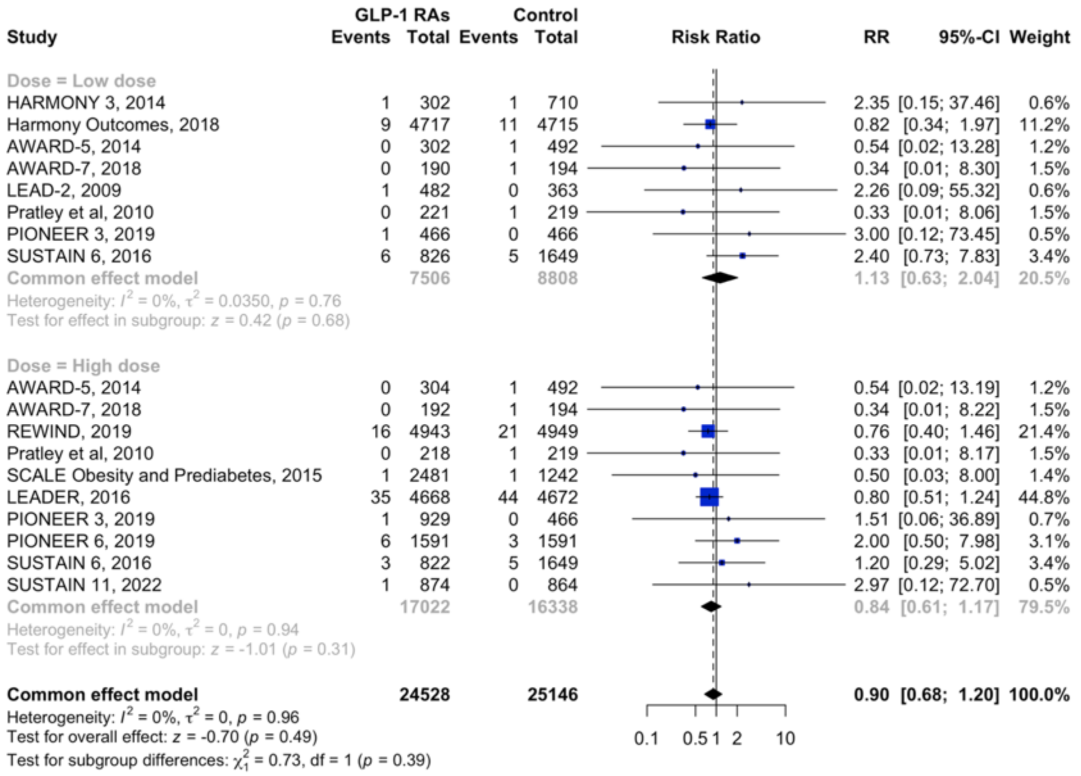

Figure S6.3. Effects of follow-up duration on the association between GLP-1 RAs and the risk of sudden cardiac death

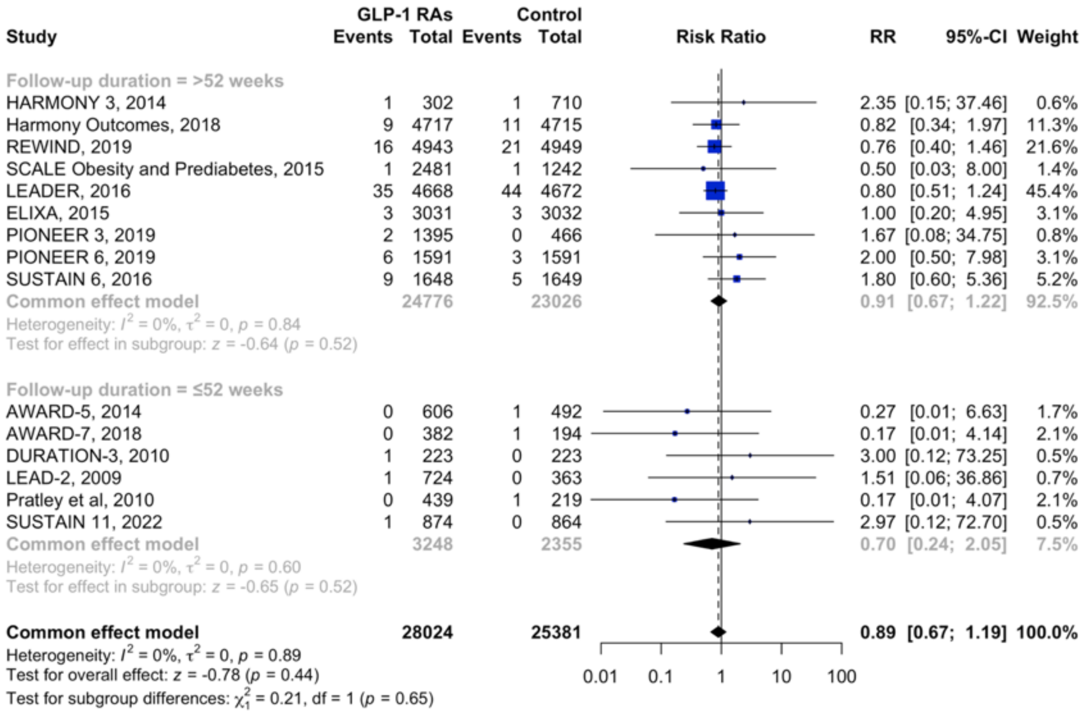

Figure S6.4. Effects of baseline BMI on the association between GLP-1 RAs and risks of sudden cardiac death

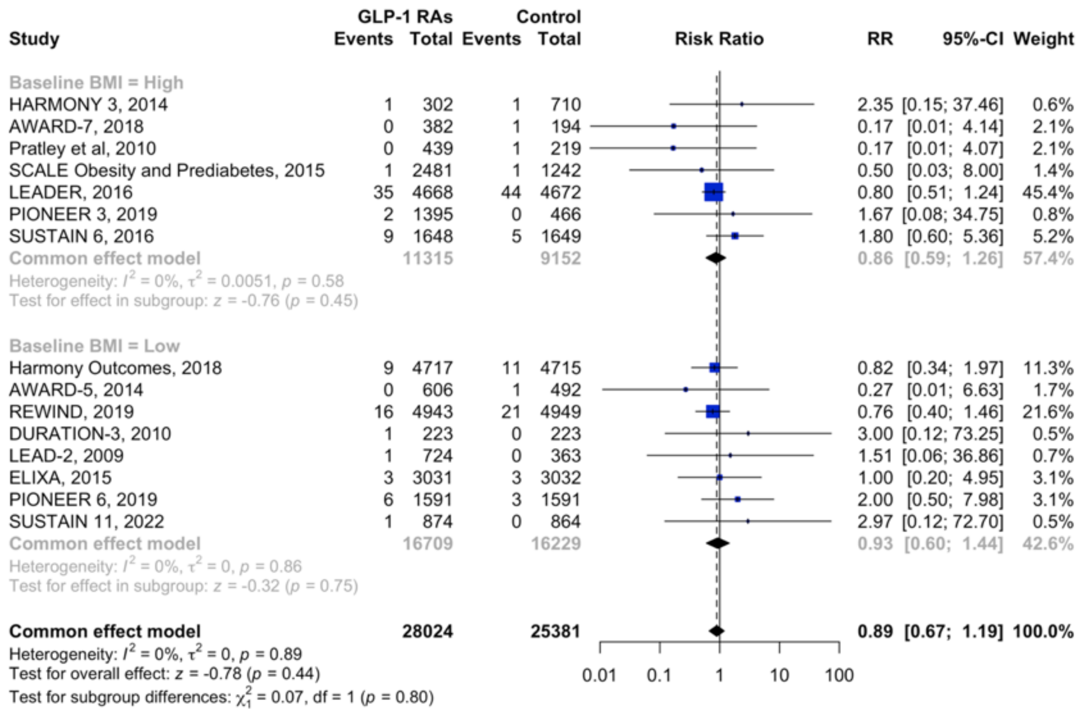

Figure S6.5. Effects of study designs on the association between GLP-1 RAs and risks of sudden cardiac death

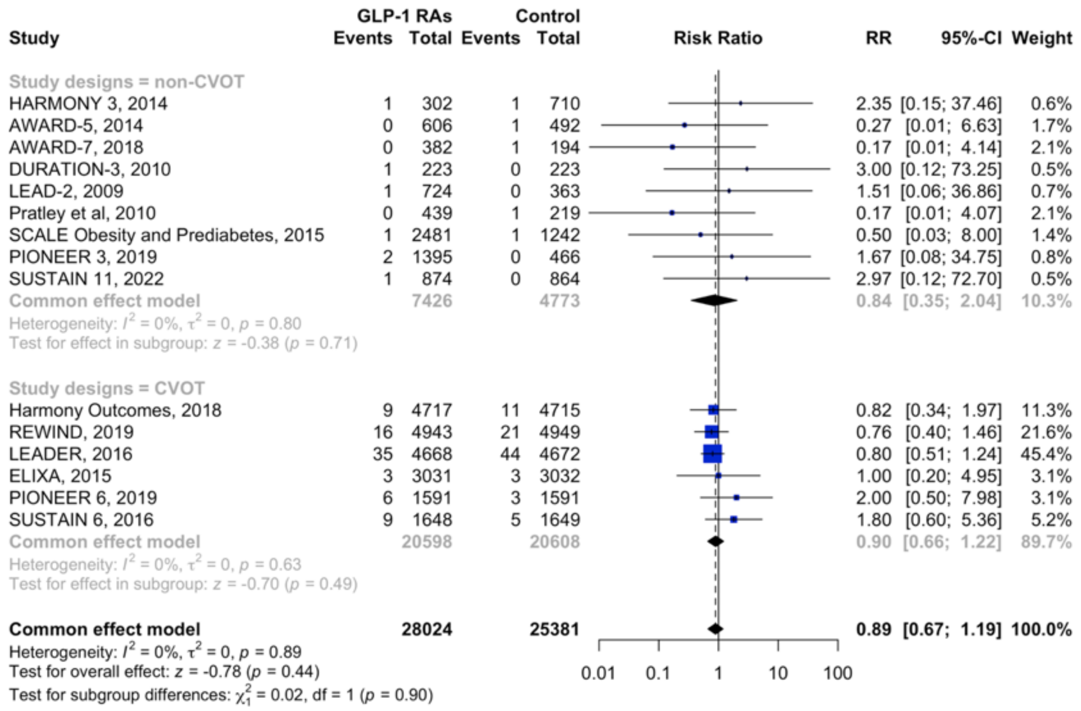

Figure S7. Sensitivity analyses by omitting each trial one by one of all included studies

Notes: Experimental, GLP-1RAs treatments; Control, placebo or active control. Abbreviations: RR, relative risks; CI, confidential intervals.

Figure S7.1. Sensitivity analysis of all included studies on atrial fibrillation

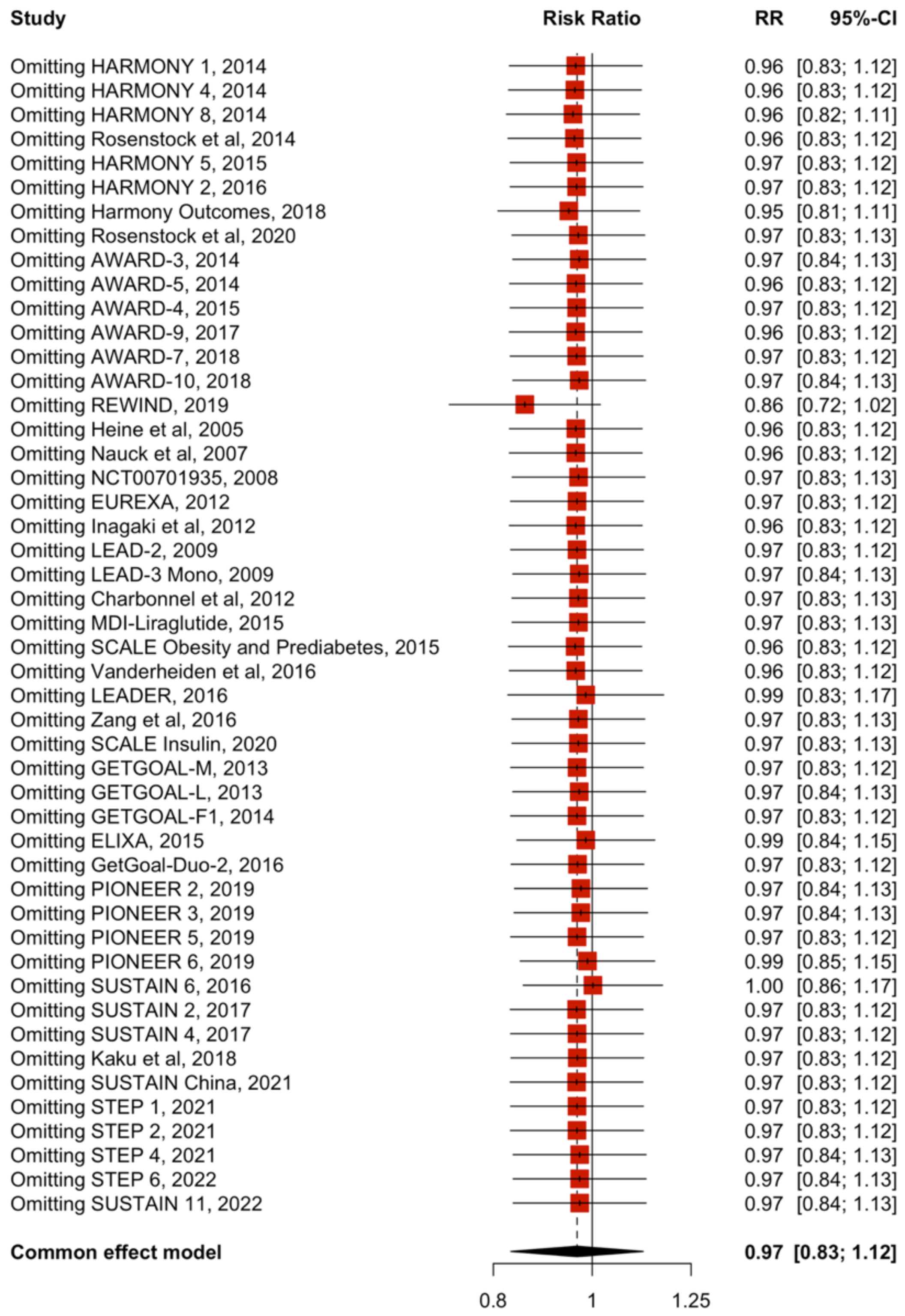

Figure S7.2. Sensitivity analysis of all included studies on the association between GLP-1RAs and the risk of atrial flutter

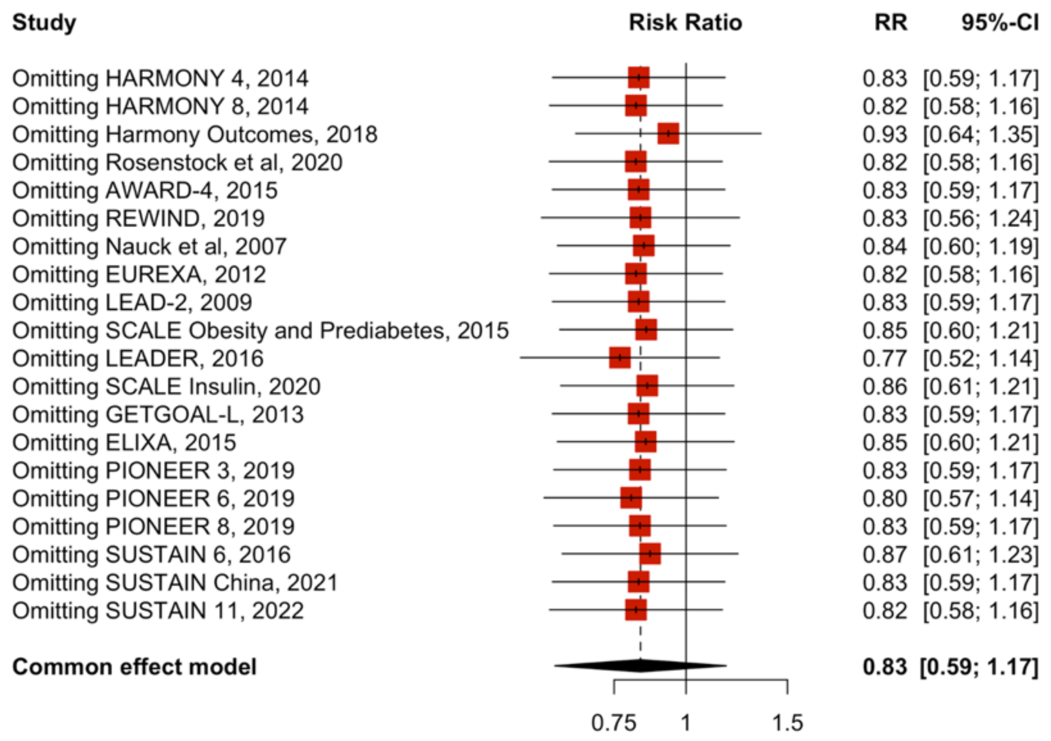

Figure S7.3. Sensitivity analysis of all included studies on the association between GLP-1RAs and the risk of ventricular arrhythmias

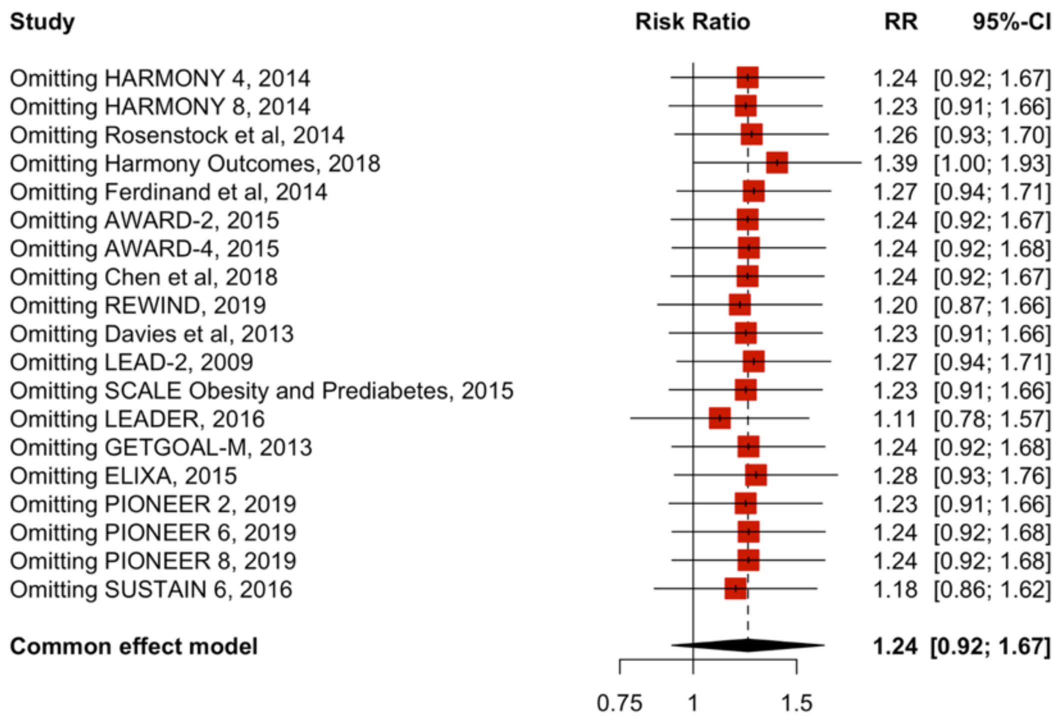

Figure S7.4. Sensitivity analysis of all included studies on the association between GLP-1RAs and the risk of sudden cardiac death

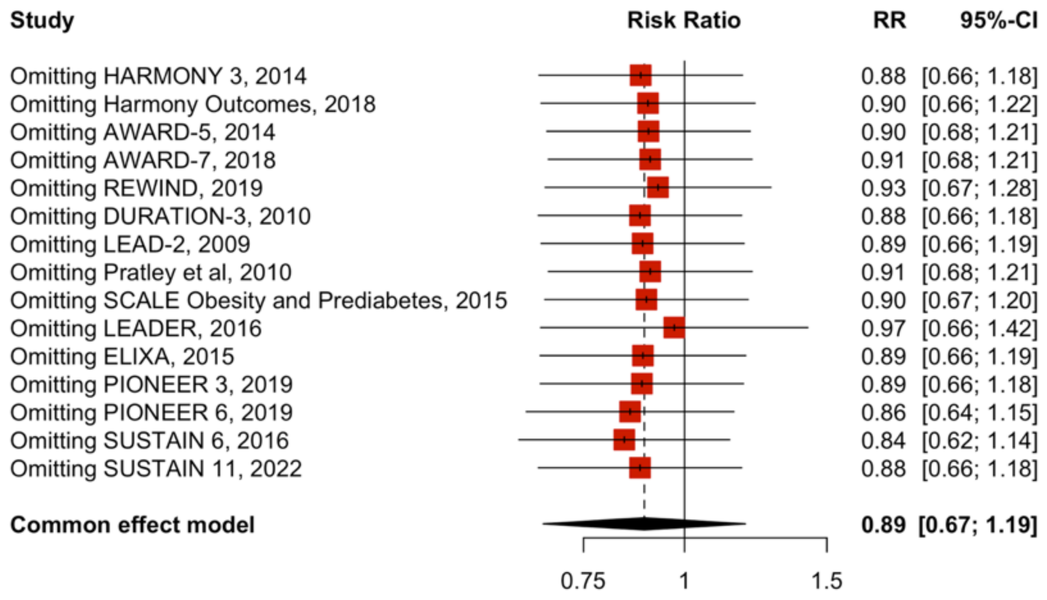

Figure S7.5. Sensitivity analysis of studies on the association between dulaglutide and the risk of atrial fibrillation

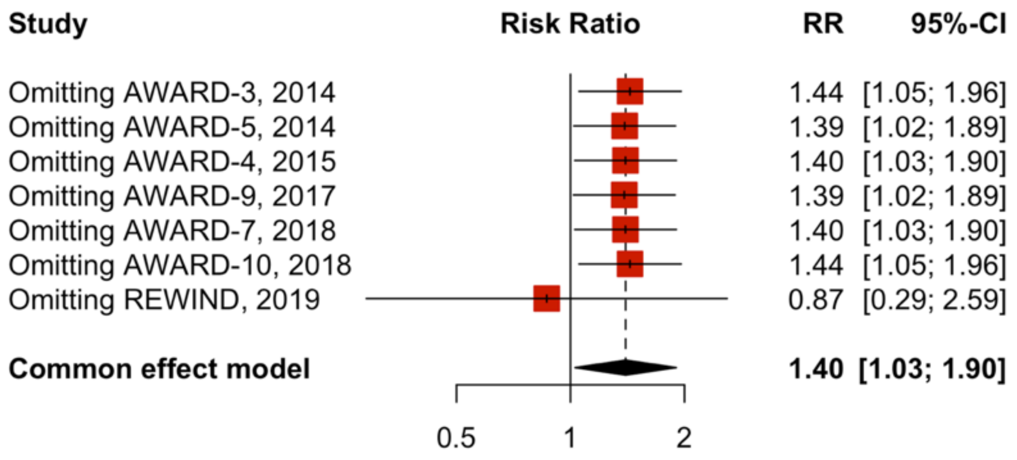

Figure S7.6. Sensitivity analysis of studies on the association between oral semaglutide and the risk of atrial fibrillation

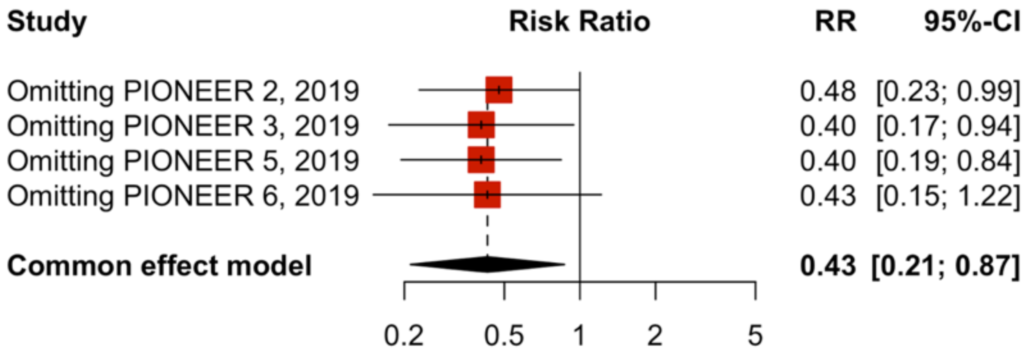

Figure S7.7. Sensitivity analysis of studies about the association between high dose of GLP-1RAs and the risk of ventricular arrhythmias

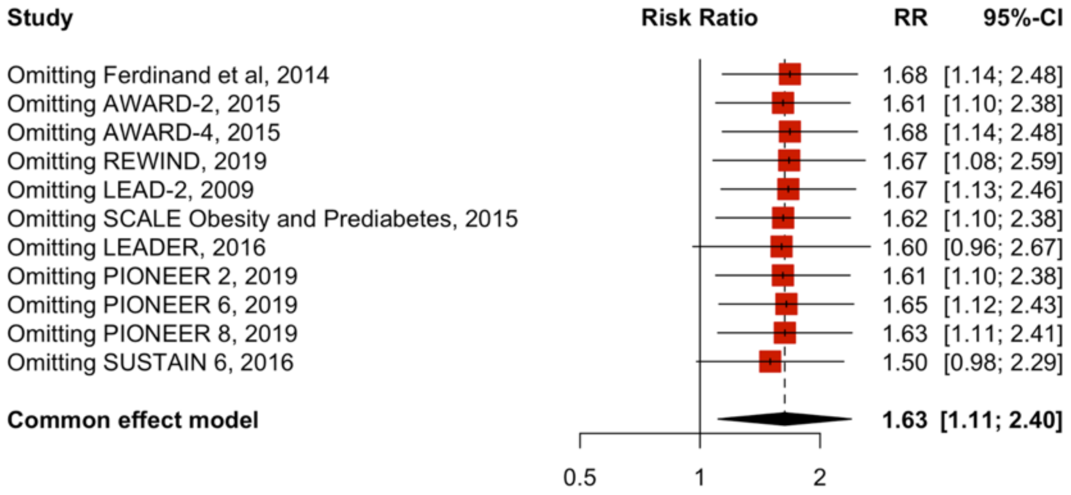

Figure S7.8. Sensitivity analysis of studies about the association between high baseline BMI and the risk of ventricular arrhythmias.

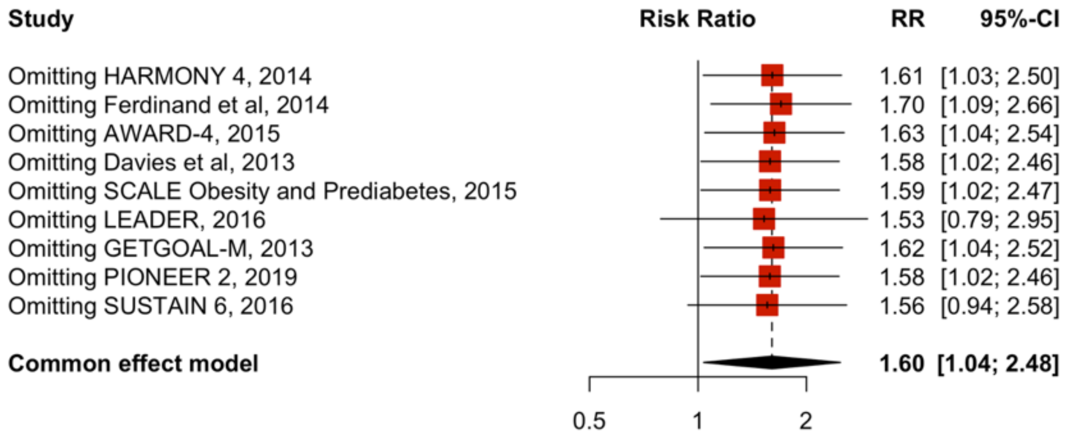

Figure S8. Funnel plots for each outcome

Abbreviations: GLP-1RAs, glucagon-like peptide 1 receptor agonists.

Figure S8.1. Funnel plot of studies included for the association between GLP-1RAs and the risk of atrial fibrillation

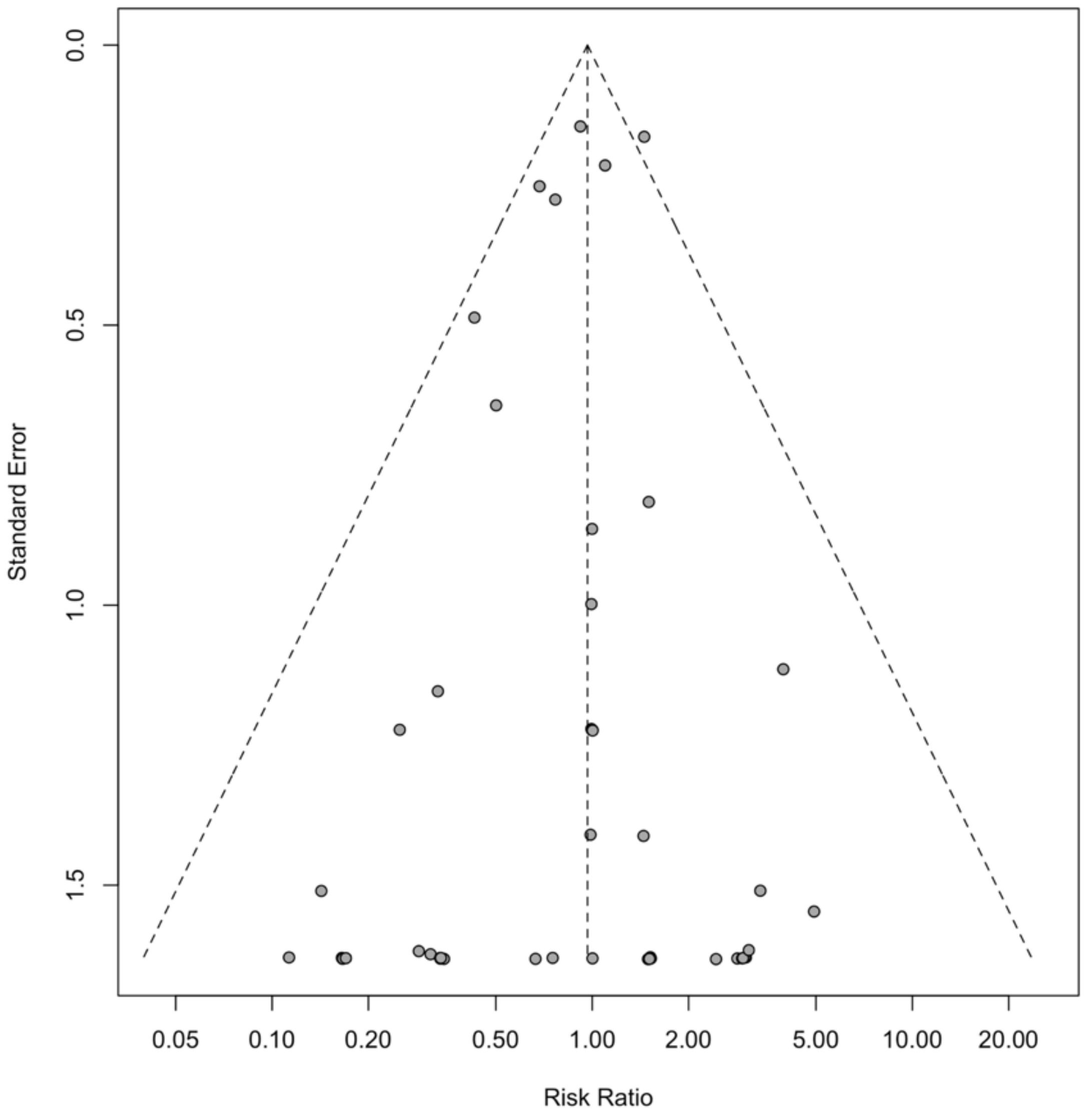

Figure S8.2. Funnel plot of studies included for the association between GLP-1RAs and the risk of atrial flutter

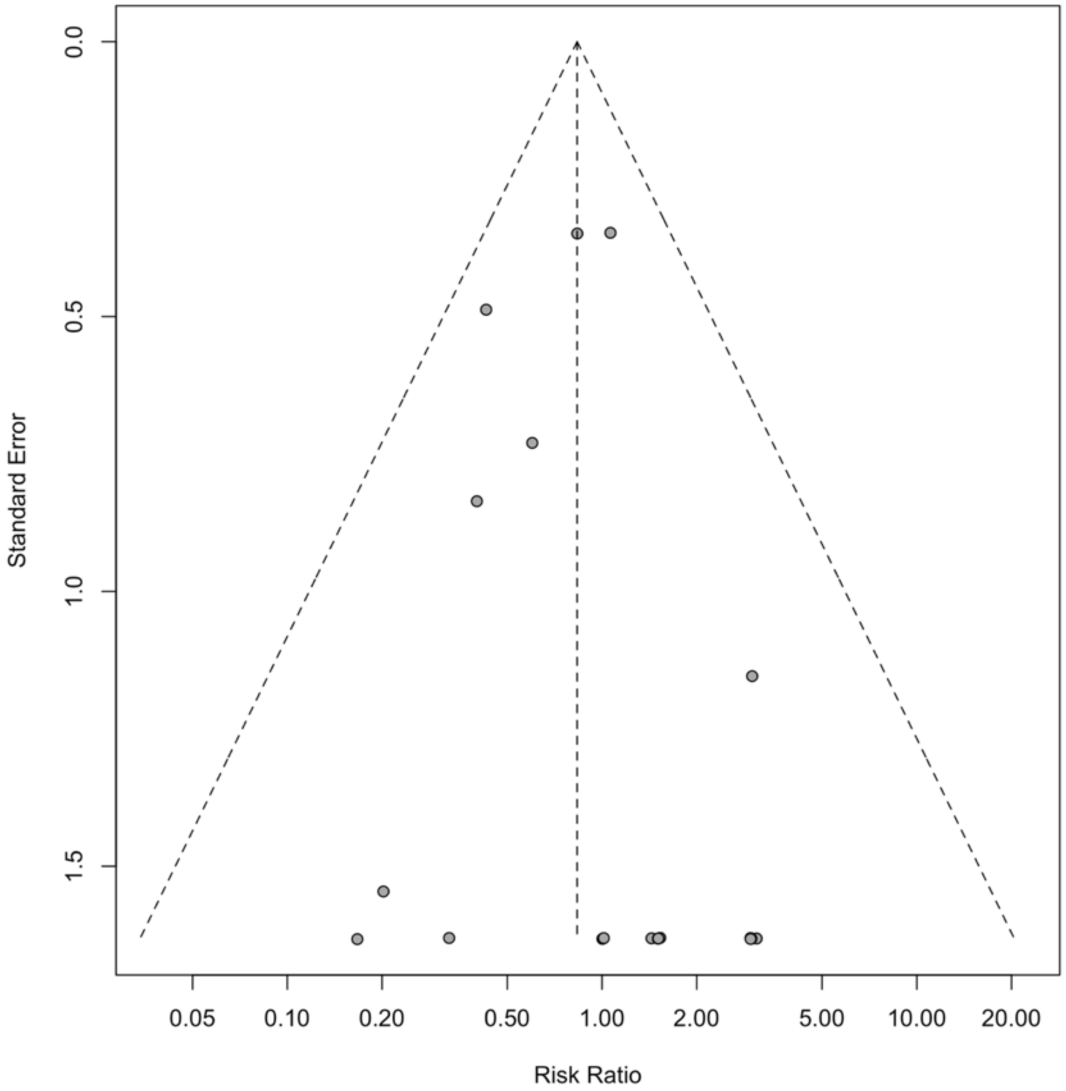

Figure S8.3. Funnel plot of studies included for the association between GLP-1RAs and the risk of ventricular arrhythmias

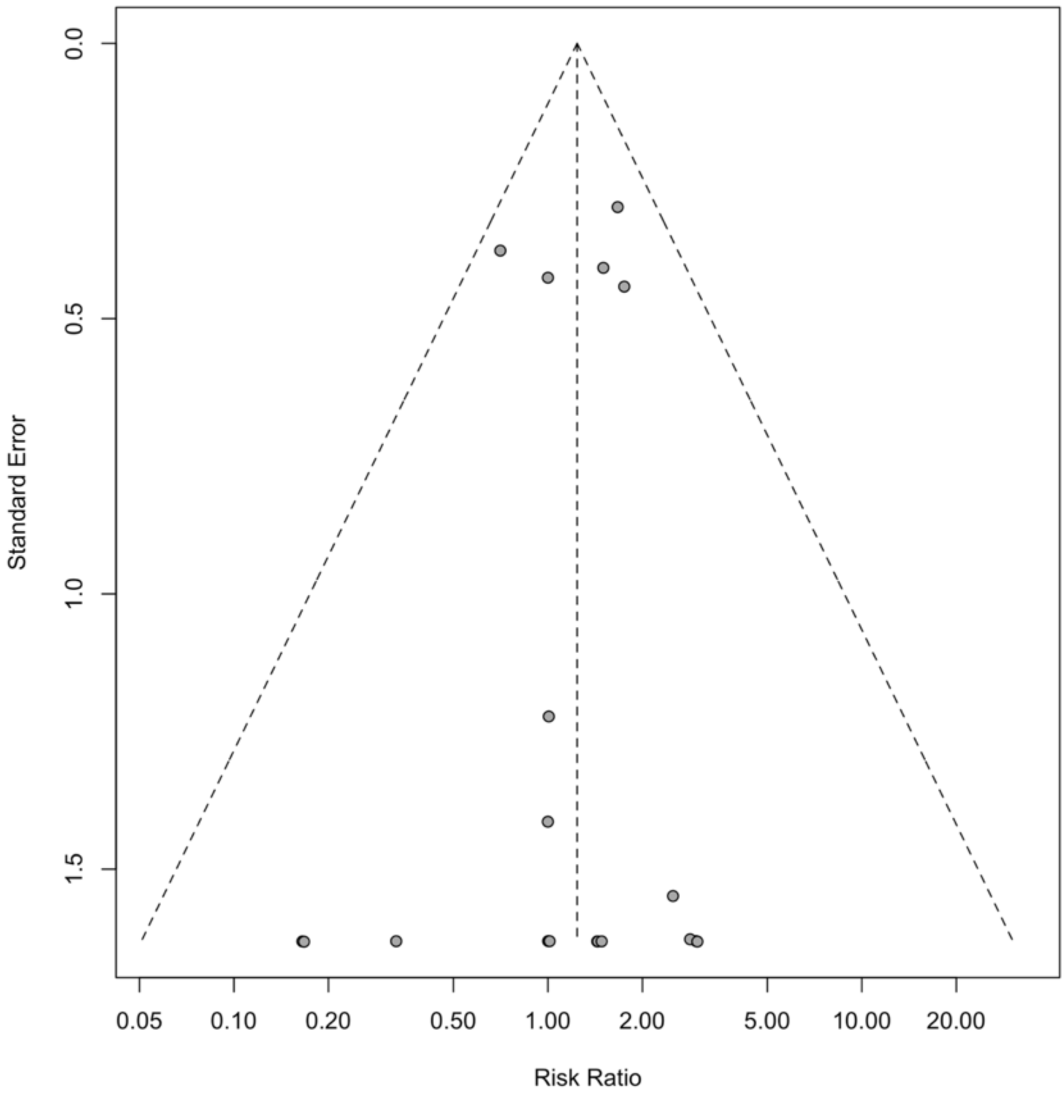

Figure S8.4. Funnel plot of studies included for the association between GLP-1RAs and the risks of sudden cardiac death

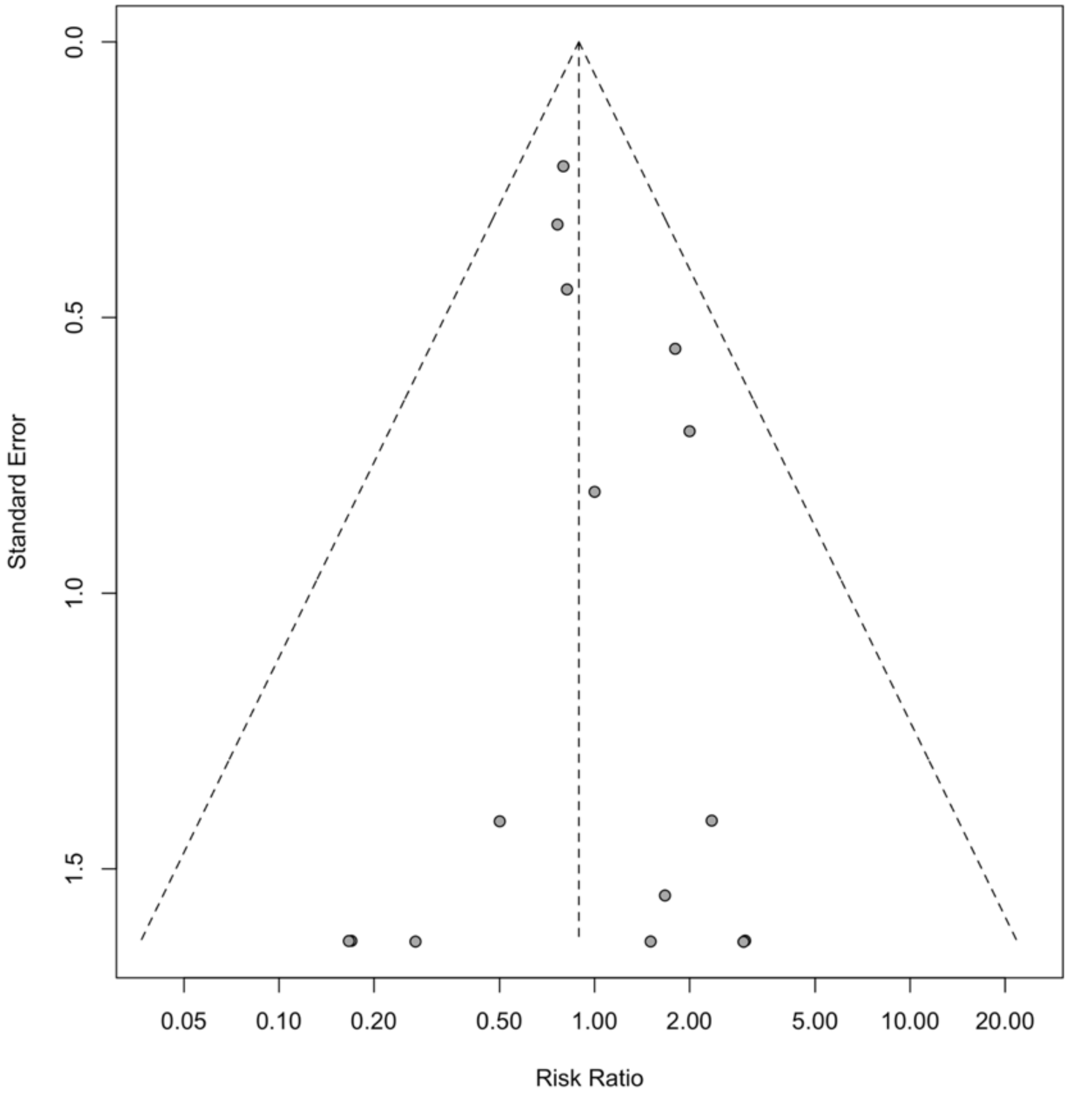

References S1

1. Reusch J, Stewart MW, Perkins CM, Cirkel DT, Ye J, et al. Efficacy and safety of once-weekly glucagon-like peptide 1 receptor agonist albiglutide (HARMONY 1 trial): 52-week primary endpoint results from a randomized, double-blind, placebo-controlled trial in patients with type 2 diabetes mellitus not controlled on pioglitazone, with or without metformin. Diabetes Obes Metab. 2014;16:1257-64.
2. Ahrén B, Johnson SL, Stewart M, Cirkel DT, Yang F, et al. HARMONY 3: 104-week randomized, double-blind, placebo- and active-controlled trial assessing the efficacy and safety of albiglutide compared with placebo, sitagliptin, and glimepiride in patients with type 2 diabetes taking metformin. Diabetes Care. 2014;37:2141-8.
3. Weissman PN, Carr MC, Ye J, Cirkel DT, Stewart M, et al. HARMONY 4: randomised clinical trial comparing once-weekly albiglutide and insulin glargine in patients with type 2 diabetes inadequately controlled with metformin with or without sulfonylurea. Diabetologia. 2014;57:2475-84.
4. Leiter LA, Carr MC, Stewart M, Jones-Leone A, Scott R, et al. Efficacy and safety of the once-weekly GLP-1 receptor agonist albiglutide versus sitagliptin in patients with type 2 diabetes and renal impairment: a randomized phase III study. Diabetes Care. 2014;37:2723-30.
5. Rosenstock J, Fonseca VA, Gross JL, Ratner RE, Ahrén B, et al. Advancing basal insulin replacement in type 2 diabetes inadequately controlled with insulin glargine plus oral agents: A comparison of adding albiglutide, a weekly GLP-1 receptor agonist, versus thrice-daily prandial insulin lispro. Diabetes Care. 2014;37:2317-25.
6. Home PD, Shamanna P, Stewart M, Yang F, Miller M, et al. Efficacy and tolerability of albiglutide versus placebo or pioglitazone over 1year in people with type 2 diabetes currently taking metformin and glimepiride: HARMONY 5. Diabetes Obes Metab. 2015;17:179-87.
7. Nauck MA, Stewart MW, Perkins C, Jones-Leone A, Yang F, et al. Efficacy and safety of once-weekly GLP-1 receptor agonist albiglutide (HARMONY 2):

52 week primary endpoint results from a randomised, placebo-controlled trial in patients with type 2 diabetes mellitus inadequately controlled with diet and exercise. Diabetologia. 2016;59:266-74.

8. Hernandez AF, Green JB, Janmohamed S, D'Agostino RB, Sr., Granger CB, et al. Albiglutide and cardiovascular outcomes in patients with type 2 diabetes and cardiovascular disease (Harmony Outcomes): a double-blind, randomised placebo-controlled trial. Lancet. 2018;392:1519-29.
9. Rosenstock J, Nino A, Soffer J, Erskine L, Acusta A, et al. Impact of a Weekly Glucagon-Like Peptide 1 Receptor Agonist, Albiglutide, on Glycemic Control and on Reducing Prandial Insulin Use in Type 2 Diabetes Inadequately Controlled on Multiple Insulin Therapy: A Randomized Trial. Diabetes Care. 2020;43:2509-18.
10. Ferdinand KC, White WB, Calhoun DA, Lonn EM, Sager PT, et al. Effects of the once-weekly glucagon-like peptide-1 receptor agonist dulaglutide on ambulatory blood pressure and heart rate in patients with type 2 diabetes mellitus. Hypertension. 2014;64:731-7.
11. Umpierrez G, Tofé Povedano S, Pérez Manghi F, Shurzinske L, Pechtner V. Efficacy and safety of dulaglutide monotherapy versus metformin in type 2 diabetes in a randomized controlled trial (AWARD-3). Diabetes Care. 2014;37:2168-76.
12. Nauck M, Weinstock RS, Umpierrez GE, Guerci B, Skrivanek Z, et al. Efficacy and safety of dulaglutide versus sitagliptin after 52 weeks in type 2 diabetes in a randomized controlled trial (AWARD-5) Diabetes Care. 2014;37:2149-58.
13. Giorgino F, Benroubi M, Sun JH, Zimmermann AG, Pechtner V. Efficacy and Safety of Once-Weekly Dulaglutide Versus Insulin Glargine in Patients With Type 2 Diabetes on Metformin and Glimepiride (AWARD-2). Diabetes Care. 2015;38:2241-9.
14. Blonde L, Jendle J, Gross J, Woo V, Jiang H, et al. Once-weekly dulaglutide versus bedtime insulin glargine, both in combination with prandial insulin lispro, in patients with type 2 diabetes (AWARD-4): a randomised, open-label, phase 3, non-inferiority study. Lancet. 2015;385:2057-66.

15. Pozzilli P, Norwood P, Jódar E, Davies MJ, Ivanyi T, et al. Placebo-controlled, randomized trial of the addition of once-weekly glucagon-like peptide-1 receptor agonist dulaglutide to titrated daily insulin glargine in patients with type 2 diabetes (AWARD-9). Diabetes Obes Metab. 2017;19:1024-31.
16. Tuttle KR, Lakshmanan MC, Rayner B, Busch RS, Zimmermann AG, et al. Dulaglutide versus insulin glargine in patients with type 2 diabetes and moderate-to-severe chronic kidney disease (AWARD-7): a multicentre, open-label, randomised trial. Lancet Diabetes Endocrinol. 2018;6:605-17.
17. Ludvik B, Frías JP, Tinahones FJ, Wainstein J, Jiang H, et al. Dulaglutide as add-on therapy to SGLT2 inhibitors in patients with inadequately controlled type 2 diabetes (AWARD-10): a 24-week, randomised, double-blind, placebo-controlled trial. Lancet Diabetes Endocrinol. 2018;6:370-81.
18. Chen YH, Huang CN, Cho YM, Li P, Gu L, et al. Efficacy and safety of dulaglutide monotherapy compared with glimepiride in East-Asian patients with type 2 diabetes in a multicentre, double-blind, randomized, parallel-arm, active comparator, phase III trial. Diabetes Obes Metab. 2018;20:2121-30.
19. Gerstein HC, Colhoun HM, Dagenais GR, Diaz R, Lakshmanan M, et al. Dulaglutide and cardiovascular outcomes in type 2 diabetes (REWIND): a double-blind, randomised placebo-controlled trial. Lancet. 2019;394:121-30.
20. Heine RJ, Van Gaal LF, Johns D, Mihm MJ, Widel MH, et al. Exenatide versus insulin glargine in patients with suboptimally controlled type 2 diabetes: a randomized trial. Ann Intern Med. 2005;143:559-69.
21. Nauck MA, Duran S, Kim D, Johns D, Northrup J, et al. A comparison of twice-daily exenatide and biphasic insulin aspart in patients with type 2 diabetes who were suboptimally controlled with sulfonylurea and metformin: a non-inferiority study. Diabetologia. 2007;50:259-67.
22. AstraZeneca, Lilly E, Company. Effect of Exenatide on Abdominal Fat Distribution in Patients With Type 2 Diabetes Pretreated With Metformin. In.: https://ClinicalTrials.gov/show/NCT00701935; 2008.
23. Gallwitz B, Guzman J, Dotta F, Guerci B, Simó R, et al. Exenatide twice daily

versus glimepiride for prevention of glycaemic deterioration in patients with type 2 diabetes with metformin failure (EUREXA): an open-label, randomised controlled trial. Lancet. 2012;379:2270-8.
24. Diamant M, Van Gaal L, Stranks S, Northrup J, Cao D, et al. Once weekly exenatide compared with insulin glargine titrated to target in patients with type 2 diabetes (DURATION-3): an open-label randomised trial. Lancet. 2010;375:2234-43.
25. Inagaki N, Atsumi Y, Oura T, Saito H, Imaoka T. Efficacy and safety profile of exenatide once weekly compared with insulin once daily in Japanese patients with type 2 diabetes treated with oral antidiabetes drug(s): results from a 26-week, randomized, open-label, parallel-group, multicenter, noninferiority study. Clin Ther. 2012;34:1892-908.e1.
26. Davies M, Heller S, Sreenan S, Sapin H, Adetunji O, et al. Once-weekly exenatide versus once- or twice-daily insulin detemir: randomized, open-label, clinical trial of efficacy and safety in patients with type 2 diabetes treated with metformin alone or in combination with sulfonylureas. Diabetes Care. 2013;36:1368-76.
27. Nauck M, Frid A, Hermansen K, Shah NS, Tankova T, et al. Efficacy and safety comparison of liraglutide, glimepiride, and placebo, all in combination with metformin, in type 2 diabetes. Diabetes Care. 2009;32:84-90.
28. Garber A, Henry R, Ratner R, Garcia-Hernandez PA, Rodriguez-Pattzi H, et al. Liraglutide versus glimepiride monotherapy for type 2 diabetes (LEAD-3 Mono): a randomised, 52-week, phase III, double-blind, parallel-treatment trial. Lancet. 2009;373:473-81.
29. Pratley RE, Nauck M, Bailey T, Montanya E, Cuddihy R, et al. Liraglutide versus sitagliptin for patients with type 2 diabetes who did not have adequate glycaemic control with metformin: a 26-week, randomised, parallel-group, open-label trial. Lancet. 2010;375:1447-56.
30. Charbonnel B, Steinberg H, Eymard E, Xu L, Thakkar P, et al. Efficacy and safety over 26 weeks of an oral treatment strategy including sitagliptin compared with an injectable treatment strategy with liraglutide in patients with type 2 diabetes

mellitus inadequately controlled on metformin: a randomised clinical trial. Diabetologia. 2013;56:1503-11.

31. Lind M, Hirsch IB, Tuomilehto J, Dahlqvist S, Ahrén B, et al. Liraglutide in people treated for type 2 diabetes with multiple daily insulin injections: Randomised clinical trial (MDI Liraglutide trial). BMJ. 2015;351.
32. Pi-Sunyer X, Astrup A, Fujioka K, Greenway F, Halpern A, et al. A randomized, controlled trial of 3.0 mg of liraglutide in weight management. New England Journal of Medicine. 2015;373:11-22.
33. Vanderheiden A, Harrison L, Warshauer J, Li X, Adams-Huet B, et al. Effect of Adding Liraglutide vs Placebo to a High-Dose lnsulin Regimen in Patients With Type 2 Diabetes: A Randomized Clinical Trial. JAMA Intern Med. 2016;176:939-47.
34. Marso SP, Daniels GH, Brown-Frandsen K, Kristensen P, Mann JF, et al. Liraglutide and Cardiovascular Outcomes in Type 2 Diabetes. N Engl J Med. 2016;375:311-22.
35. Zang L, Liu Y, Geng J, Luo Y, Bian F, et al. Efficacy and safety of liraglutide versus sitagliptin, both in combination with metformin, in Chinese patients with type 2 diabetes: a 26-week, open-label, randomized, active comparator clinical trial. Diabetes Obes Metab. 2016;18:803-11.
36. Garvey WT, Birkenfeld AL, Dicker D, Mingrone G, Pedersen SD, et al. Efficacy and Safety of Liraglutide 3.0 mg in Individuals With Overweight or Obesity and Type 2 Diabetes Treated With Basal Insulin: The SCALE Insulin Randomized Controlled Trial. Diabetes Care. 2020;43:1085-93.
37. Ahrén B, Leguizamo Dimas A, Miossec P, Saubadu S, Aronson R. Efficacy and safety of lixisenatide once-daily morning or evening injections in type 2 diabetes inadequately controlled on metformin (GetGoal-M). Diabetes Care. 2013;36:2543-50.
38. Riddle MC, Aronson R, Home P, Marre M, Niemoeller E, et al. Adding once-daily lixisenatide for type 2 diabetes inadequately controlled by established basal insulin: a 24-week, randomized, placebo-controlled comparison (GetGoal-L). Diabetes

Care. 2013;36:2489-96.

39. Bolli GB, Munteanu M, Dotsenko S, Niemoeller E, Boka G, et al. Efficacy and safety of lixisenatide once daily vs. placebo in people with Type 2 diabetes insufficiently controlled on metformin (GetGoal-F1). Diabet Med. 2014;31:176-84.
40. Pfeffer MA, Claggett B, Diaz R, Dickstein K, Gerstein HC, et al. Lixisenatide in patients with type 2 diabetes and acute coronary syndrome. N Engl J Med. 2015;373:2247-57.
41. Rosenstock J, Guerci B, Hanefeld M, Gentile S, Aronson R, et al. Prandial Options to Advance Basal Insulin Glargine Therapy: Testing Lixisenatide Plus Basal Insulin Versus Insulin Glulisine Either as Basal-Plus or Basal-Bolus in Type 2 Diabetes: The GetGoal Duo-2 Trial. Diabetes Care. 2016;39:1318-28.
42. Rodbard HW, Rosenstock J, Canani LH, Deerochanawong C, Gumprecht J, et al. Oral semaglutide versus empagliflozin in patients with type 2 diabetes uncontrolled on metformin: The PIONEER 2 trial. Diabetes Care. 2019;42:2272-81.
43. Rosenstock J, Allison D, Birkenfeld AL, Blicher TM, Deenadayalan S, et al. Effect of Additional Oral Semaglutide vs Sitagliptin on Glycated Hemoglobin in Adults With Type 2 Diabetes Uncontrolled With Metformin Alone or With Sulfonylurea: The PIONEER 3 Randomized Clinical Trial. JAMA. 2019;321:1466-80.
44. Mosenzon O, Blicher TM, Rosenlund S, Eriksson JW, Heller S, et al. Efficacy and safety of oral semaglutide in patients with type 2 diabetes and moderate renal impairment (PIONEER 5): a placebo-controlled, randomised, phase 3a trial. Lancet Diabetes Endocrinol. 2019;7:515-27.
45. Husain M, Birkenfeld AL, Donsmark M, Dungan K, Eliaschewitz FG, et al. Oral Semaglutide and Cardiovascular Outcomes in Patients with Type 2 Diabetes. N Engl J Med. 2019;381:841-51.
46. Zinman B, Aroda VR, Buse JB, Cariou B, Harris SB, et al. Efficacy, Safety, and Tolerability of Oral Semaglutide Versus Placebo Added to Insulin With or Without Metformin in Patients With Type 2 Diabetes: The PIONEER 8 Trial

NCT03021187. Diabetes Care. 2019;42:2262-71.
47. Marso SP, Bain SC, Consoli A, Eliaschewitz FG, Jódar E, et al. Semaglutide and Cardiovascular Outcomes in Patients with Type 2 Diabetes. N Engl J Med. 2016;375:1834-44.
48. Ahrén B, Masmiquel L, Kumar H, Sargin M, Karsbøl JD, et al. Efficacy and safety of once-weekly semaglutide versus once-daily sitagliptin as an add-on to metformin, thiazolidinediones, or both, in patients with type 2 diabetes (SUSTAIN 2): a 56-week, double-blind, phase 3a, randomised trial. Lancet Diabetes Endocrinol. 2017;5:341-54.
49. Aroda VR, Bain SC, Cariou B, Piletič M, Rose L, et al. Efficacy and safety of once-weekly semaglutide versus once-daily insulin glargine as add-on to metformin (with or without sulfonylureas) in insulin-naive patients with type 2 diabetes (SUSTAIN 4): a randomised, open-label, parallel-group, multicentre, multinational, phase 3a trial. Lancet Diabetes Endocrinol. 2017;5:355-66.
50. Kaku K, Yamada Y, Watada H, Abiko A, Nishida T, et al. Safety and efficacy of once-weekly semaglutide vs additional oral antidiabetic drugs in Japanese people with inadequately controlled type 2 diabetes: A randomized trial. Diabetes Obes Metab. 2018;20:1202-12.
51. Ji L, Dong X, Li Y, Li Y, Lim S, et al. Efficacy and safety of once-weekly semaglutide versus once-daily sitagliptin as add-on to metformin in patients with type 2 diabetes in SUSTAIN China: A 30-week, double-blind, phase 3a, randomized trial. Diabetes Obes Metab. 2021;23:404-14.
52. Wilding JPH, Batterham RL, Calanna S, Davies M, Van Gaal LF, et al. Once-Weekly Semaglutide in Adults with Overweight or Obesity. N Engl J Med. 2021;384:989-1002.
53. Davies M, Færch L, Jeppesen OK, Pakseresht A, Pedersen SD, et al. Semaglutide 2·4 mg once a week in adults with overweight or obesity, and type 2 diabetes (STEP 2): a randomised, double-blind, double-dummy, placebo-controlled, phase 3 trial. Lancet. 2021;397:971-84.
54. Rubino D, Abrahamsson N, Davies M, Hesse D, Greenway FL, et al. Effect of

Continued Weekly Subcutaneous Semaglutide vs Placebo on Weight Loss Maintenance in Adults With Overweight or Obesity: The STEP 4 Randomized Clinical Trial. JAMA. 2021;325:1414-25.

55. Kadowaki T, Isendahl J, Khalid U, Lee SY, Nishida T, et al. Semaglutide once a week in adults with overweight or obesity, with or without type 2 diabetes in an east Asian population (STEP 6): a randomised, double-blind, double-dummy, placebo-controlled, phase 3a trial. Lancet Diabetes Endocrinol. 2022;10:193-206.
56. Kellerer M, Kaltoft MS, Lawson J, Nielsen LL, Strojek K, et al. Effect of once-weekly semaglutide versus thrice-daily insulin aspart, both as add-on to metformin and optimized insulin glargine treatment in participants with type 2 diabetes (SUSTAIN 11): a randomized, open-label, multinational, phase 3b trial. Diabetes Obes Metab. 2022.
